# Targeted Therapeutic Nanoparticles: An Immense Promise to Fight against Cancer

**DOI:** 10.1155/2017/9090325

**Published:** 2017-12-31

**Authors:** Sheikh Tasnim Jahan, Sams M. A. Sadat, Matthew Walliser, Azita Haddadi

**Affiliations:** Division of Pharmacy, College of Pharmacy and Nutrition, University of Saskatchewan, Saskatoon, SK, Canada S7N 5E5

## Abstract

In nanomedicine, targeted therapeutic nanoparticle (NP) is a virtual outcome of nanotechnology taking the advantage of cancer propagation pattern. Tying up all elements such as therapeutic or imaging agent, targeting ligand, and cross-linking agent with the NPs is the key concept to deliver the payload selectively where it intends to reach. The microenvironment of tumor tissues in lymphatic vessels can also help targeted NPs to achieve their anticipated accumulation depending on the formulation objectives. This review accumulates the application of poly(lactic-co-glycolic acid) (PLGA) and polyethylene glycol (PEG) based NP systems, with a specific perspective in cancer. Nowadays, PLGA, PEG, or their combinations are the mostly used polymers to serve the purpose of targeted therapeutic NPs. Their unique physicochemical properties along with their biological activities are also discussed. Depending on the biological effects from parameters associated with existing NPs, several advantages and limitations have been explored in teaming up all the essential facts to give birth to targeted therapeutic NPs. Therefore, the current article will provide a comprehensive review of various approaches to fabricate a targeted system to achieve appropriate physicochemical properties. Based on such findings, researchers can realize the benefits and challenges for the next generation of delivery systems.

## 1. Introduction

Applied nanotechnology is a revolutionary field with immense potential owing to recent advancements in NP-based drug delivery systems. In general, colloidal NPs are the creates that possess physicochemical features with a size range of 1–1000 nm [1]. The aim of most nanodevices is to prevent the degradation of active molecules to have enhanced bioavailability and to regulate their pharmacokinetic profile. However, most drugs are associated with some limitations such as poor water solubility, improper size and surface area, biodistribution and targeting challenges, and low therapeutic index [2]. To overcome these shortcomings, scientists are always in search for the improved, structurally stable therapeutic NPs that offer several advantages over the free drug. The NPs generally offer enormous surface area, high drug loading capacity, feasibility of functionalization with ligands, controlled drug-release capacity, minimal toxicity, biocompatibility, storage stability, and flexibility in the route of administration. Despite the advantages offered by NPs, the challenges associated should be considered before formulating any therapeutic NPs; some of the challenges are as follows [3, 4]:

- Nontargeted NPs could easily be recognized by mononuclear phagocyte system (MPS) present in blood, liver, spleen, lung, and bone marrow.
- Surface hydrophobicity of NPs is a key factor for enhanced blood components adsorption onto the NP surface.
- Prolonged circulation time of NPs is a prerequisite for* in vivo* administration until they reach at the target site.
- Localization of NPs to the tumor following enhanced permeability and retention (EPR) effect could be hindered by abnormal tumor structure leading to ineffective drug uptake.

Surface modification of the NPs with suitable targeting moieties could overcome these challenges to some extent since targeting agents would efficiently carry the active molecule to its specific site of action rather than undesired localization [5]. This concept triggered the development of several approaches for structural modification of NPs. In this regard, numerous drug delivery systems have been tested to deliver the drug in both* in vitro* and* in vivo *models to assess their targeting efficiency. To conquer this challenge, polymeric biodegradable NPs have gained the most attraction over the past few decades for drug delivery. Among all, PLGA, PEG, polylactic acid (PLA), chitosan, gelatin, and polycaprolactone (PCL) are the widely used polymers [6]. In this review, the main focus has been given mostly for the drug delivery systems with PLGA and PEG due to their wide acceptance as a biodegradable and biocompatible polymer [7]. Depending on the preparation methods, NPs possess different definitive properties and release characteristics for the delivery of therapeutic agent [8, 9]. Although structural alterations of these NPs may control the reticuloendothelial system (RES) recognition via stealth mechanism, the efficacy of targeted NPs with definite progression has not been standardized yet.

This review presents an overview of different NP targeting strategies with their pros and cons and recent challenges acknowledged by the researchers to date. Particularly, the review will focus on different targets being targeted by NPs, the possible techniques performed to conjugate NPs with targeting agents, and their biological responses in both* in vitro* and* in vivo *studies. Therefore, a detailed understanding of the structure-activity relationship of targeted NPs could be demonstrated in the field of cancer.

## 2. NP Targeting Strategies

Conventional therapies are rapidly eliminated from the body and suffer from widespread distribution into nontargeted organs and tissues [10], whereas targeted therapeutic NPs have gained promising attention through offering a “therapeutic strategy” to tackle the requirements for frequent drug administration, higher dose, and unwanted toxicities related to the conventional therapies [11]. Therefore, an improvised treatment regimen with more patient convenience has become a necessity. Ideally, targeting refers to the specific localization of NPs to a desired site rather than indiscriminate distribution throughout the body. Before being accumulated to the diseased site, these targeted NPs are required to overcome external barriers, en route barriers, and cellular barriers [12]. Effective design of an ideal delivery system is the key foundation to overcome these barriers.

**Figure 1:**
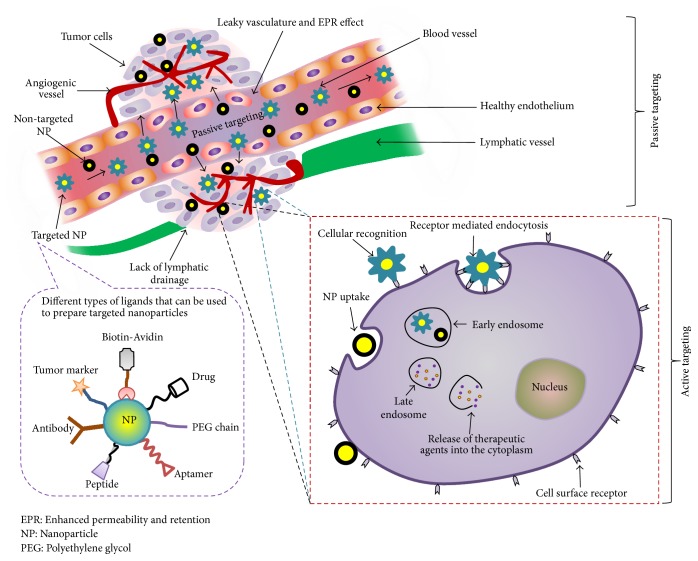
Schematic representation of passive and active targeting approaches. The diagram includes different types of ligands that can be conjugated with NPs for active targeting. EPR, enhanced permeability and retention; NP, nanoparticle; PEG, polyethylene glycol.

There are two major tumor targeting strategies, passive and active targeting, that have been widely studied as shown in Figure 1 [13]. These two strategies are correlated and work to efficiently deliver the drug particles to the target site. Passive targeting takes advantage of the pathophysiological feature of the diseased tissue, commonly tumor, while active targeting of drug carrier initially utilizes the benefits of passive targeting to accumulate into the tumor region and subsequently bind to the target cells using targeting ligand that leads to receptor mediated internalization of NPs into the cells [14].

### 2.1. Passive Targeting: Advantages and Challenges

Passive targeting depends on tumor microenvironment, EPR effect, and tumor pH to deliver therapeutic agents from the nanocarriers. It is well known that tumor cells grow and proliferate faster than normal cells. This cellular proliferation is associated with an increased metabolic rate that necessitates more nutrients and oxygen supply. In order to compete for the nutrients, the architecture of normal cells become disrupted as well as displaced by tumor cells [15]. Passive targeting allows NPs to accumulate in the neoplastic tissue through EPR effect. The normal vasculature is impermeable to molecules of size > 2–4 nm, whereas tumors have leaky vasculature facilitating the retention of NPs in the circulation due to its high density associated outer defective porous vasculature structure. In addition, the poor lymphatic drainage facilitates the stagnation of NPs within the neoplastic environment which is also an extra benefit of EPR effect [16]. Hence, passive targeting takes the advantages of the leaky vasculature as well as tumor microenvironment and helps drugs to expose directly at the tumor tissue bypassing systemic metabolism [17]. Ligand mediated active targeting could also utilize EPR effect to cross the vascular barrier [18]. Thus, EPR effect has become one of the principle considerations for the development of targeted drug delivery system.

However, EPR effect is involved with several challenges since macromolecules or NPs can invade into the tumor tissue only if they can avoid the RES and renal clearance. A drug should remain at least 6 hours in the circulation to get accumulated into the neoplastic tissues via EPR effect [19]. EPR effect was reported unsuccessful to maintain stable circulation of NPs in the bloodstream due to size restrictions of the tumor fenestrations. The development of therapeutic nanocarriers with enhanced retention time is still under practical challenge, particularly in clinical tumors, where the blood vessel morphology is very different than that of mice model used in preclinical studies. This could limit the intratumoral distribution of NPs [20]. In addition, the blunt localization and accessibility of drug carriers into the tumor might not be feasible in case of certain tumors (lung cancer). The high interstitial pressure of solid tumors does not allow homogenous distribution of drugs in the tumor. The advantages of passive targeting serve as a dilemma because EPR effect is unable to promote the uptake of NPs by target cells. In parallel, the ability of an encapsulated drug to reach their pharmacological target should also be considered. Although, passive targeting is promoting drug entry into the tumor tissue, material composition, size, and surface properties are also important determinant factors in this matter. The affinity of the drug to retain into the intratumoral environment should be considered before designing drug delivery systems for passive targeting [21]. Recently, scientists are developing NPs that can adapt the tumor microenvironment to selectively target cancer cells. There are several NP formulations in preclinical and clinical trials that are able to tackle the microenvironment through inhibiting angiogenesis, suppressing tumor growth factors, and enhancing several immune cells (T cell, NK cells, and dendritic cells) [22]. Despite the challenges, realistic clinical settings are necessary to obtain benefit of the applied NP treatment. To date, there are several FDA approved NPs such as Doxil (1995), Feridex (1996), Mylotarg (2000), Zevalin (2002), Abraxane (2005, 2013), Oncospar (2006), and Ontac (2008) that utilize EPR effect to accumulate in solid tumors [23].

#### 2.1.1. Current Candidates for Passive Targeting

*Surface Modification of NPs with PEG*. Enhanced hydrophobicity of NPs was found correlated with steric hindrance which was associated with higher particle-particle aggregation and blood opsonization [4, 24]. Poloxamers, poloxamines, and PEG are the polymers that are commonly used to increase hydrophilicity and to provide sterically stable stealth NPs [25]. Among all, PEG is the most widely applied polymer to shield the NPs from opsonization and phagocytosis [26]. PEG has the unique ability to be soluble in both aqueous and organic solvents. Therefore, it can result in activated functional end groups at one or both termini for numerous functionalities. The choice of functional groups depends on the reactivity with the hydroxyl groups of PEG. Modifying one hydroxyl group at one end, heterobifunctional PEG can be used to get linked with different macromolecules, peptides, drugs, liposomes, and so forth. However, heterobifunctional PEGs are limited due to the formation of diol, especially for the high molecular weight of PEGs [27]. Modification of the NPs surface with high molecular weight PEG leads to the stabilization of NPs dispersion, while low molecular weight PEG may result in attraction between the particle surfaces and cause instability in the dispersion [28].

Prolonged residing time of therapeutic NPs during lymphatic circulation is a predominant factor to determine their accumulation amplitude to the tumor site. PEGylation improves the residing time of PLGA NPs due to steric stabilization by avoiding MPS recognition. Consequently, relatively lower accumulation in different organs and higher accumulation in tumor cells is observed [29, 30].

PEG is an FDA approved polymer for use in the clinic. The chain length, shape, and density of PEG are also determinant factors to govern the NPs surface hydrophilicity and their uptake mechanism in biological systems [31]. One study showed that PEG-modified wheat germ agglutinin-functionalized NPs with shorter surface PEG length were associated with clathrin-mediated endocytosis, while longer surface PEG length resulted in enhanced transcytosis after 4 hours of incubation [32]. As a general observation, the size of NPs increases with increasing the chain length of PEG considered for the respective NP formulation. However, PEG-copolymer blend shows relatively opposite effect due to the amphiphilic nature of the blend [29]. Low surface coverage of NPs exhibits “mushroom” configuration and high coverage provides “brush” conformation where most PEG chains are extended away from the surface. A balance between these two configurations represents the optimal surface coverage [26]. Although PEG prolongs the NPs circulation in blood, it can hamper the release of cargo or hide the functional groups of NPs at the target site. In order to defeat these barriers, introduction of stimuli-sensitive detachable PEG can be used which might facilitate the release of cargo and able to unveil the targeting ligands or functional groups necessary to yield corresponding responses into the target microenvironment [33].

### 2.2. Active Targeting: Advantages and Challenges

Cellular recognition in molecular level could target the tumor cell actively by ligand-receptor, antigen-antibody interactions, and aptamers. Active targeting of the therapeutic drug can be accomplished by coupling drug or nanocarrier with cell-specific targeting moiety called ligands, with or without using cross-linking agents. These targeting moieties have specific affinity for the cell surface antigens (e.g., receptors) and they can differentiate between normal and tumor cells based on the receptor or antigen expression levels [34]. For example, using Herceptin targeted NPs helped to differentiate human epidermal growth factor receptor 2 (HER2) positive and HER2 negative breast cancer cells. Clearly, the active targeting of HER2 receptors on the overexpressed cells with NPs was confirmed [35].

The term “active targeting” defines a specific ligand-receptor interaction which occurs at the target site after reaching via blood circulation and extravasation. Moreover, efficient ligand-receptor interaction depends on some criteria that include availability of the receptor on the target cells and the exclusive expression of target receptors by the target cells but not in the normal healthy cells. Although surface modification could improve the circulation time, availability of sufficient amount of modified NPs around the target is still a practical challenge [36]. It is essential to realize that only a small fraction of modified NPs can reach the target, while the majority of the intravenously administered NPs are deposited in the liver and spleen with a smaller portion in the kidneys and lungs [37]. The reality of tumor targeting is not yet clinically evident to prove active targeted therapy.

Furthermore, aiming multiple surface markers is necessary to succeed in targeted drug delivery due to the fact that tumors possess a heterogeneous nature [38]. Besides, exploration of internalization mechanisms of targeted conjugates depends on the selection of targeting ligands. For targeted therapeutics, the internalization process should accumulate higher amount of drug in the tumor cells following recycling the receptor back onto the cancer cells [39, 40].

Targeting ligands would enhance receptor mediated endocytosis that will minimize the nonspecific interaction of NPs with cell membranes. The targeting efficiency of a ligand depends on its type, nature, method of loading, density, and absence/presence of PEG or spacer molecule on NP surface. It is important to design ligands based on the specificity of the antigen overexpressed on a cancerous cell surface. In addition, such antigen specific ligand tagged on outmost layer of NPs should show high affinity for their cognate receptor [104]. Concomitantly, the tumor heterogeneity and adaptability of cancers are barriers to reach an absolute active targeting. Nowadays, along with hypoxia targeted NP, magnetic, ultrasound, and temperature- and pH-sensitive nanoparticulate systems have been developed to provide additional physical stimuli in supporting active targeting based therapy [105].

In spite of the advantages of the active targeting, this approach faces some limitations as well. Mostly, the targeting ligand might expose the NP carrier system to RES. As a result, higher accumulation of NPs could occur in the unwanted organs over the expected one. Sometimes, PEG coating is employed to minimize the interaction of NPs with RES. However, the outmost ligands could shield the PEG molecules and thus prevent the NPs to circulate in lymphatic system for a long duration. In addition, this can also lead to enhanced accumulation in spleen and liver and ultimately limit the purpose of active targeted therapeutic NPs. Although, active targeting has established a toe-hold in the clinical practice, it requires some more effort to occupy the market with full-fledged successful research [106]. In a recently completed phase III trial, Vyxeos liposomes (combination of Daunorubicin and Cytarabine) have performed very efficiently against myeloid leukemia. Also, marketed product Onivyde (liposomal Irinotecan) has been administered in combination therapy against metastatic adenocarcinoma [107]. Other examples including Docetaxel loaded prostate-specific membrane antigen (PSMA) targeted polymeric NPs (phase II) and HER2 targeted liposomal Doxorubicin (phase II/III) are showing promising outcomes. To obtain clinical benefit, the active targeting with NPs may follow a disease driven approach [108].

#### 2.2.1. Available Subcellular Targets for Active Targeting

There are several targets widely studied to understand the binding capacity of targeted drug delivery system at a subcellular level. The receptors (e.g., folate receptor and peptide receptor) are the main targets that allow specific interaction of a ligand with target on the cell through their uptake via endocytosis. Phospholipid membrane of the cells is the target for the synthetic phospholipid analogs which controls the signal transduction mechanisms. Cell surface proteins or antigens are the ideal targets because of their accessibility due to overexpression in tumor cells compared to normal cells [109]. The plasma membrane is another important target because many therapeutic proteins of interest are localized in this area. For example, human immunodeficiency virus (HIV) can be targeted by a membrane anchor inhibitor; and lipidated membrane anchored peptides can inhibit hepatitis B virus entry. Moreover, both intracellular membrane and compartments are also target for drug-ligand conjugates. Ligands of the cell surface receptor (folate and transferrin) mediate the effective transfer of anticancer drug to the early endosomes to inhibit cell signaling system. Several anticancer therapies could target endosomes that have been internalized through receptor mediated endocytosis. Transferrin-drug conjugate is mainly sorted in early endosomes, within which the ligand is released from the receptor. Antigenic peptides can be conjugated to antigens present on major histocompatibility class 1 complex for delivering the antigen-peptide conjugate to the endoplasmic reticulum. Cytosolic delivery can be performed by conjugating cell penetrating peptides with various formulations. In addition, nucleus and mitochondria can also serve as targets for drug-ligand conjugate [110].

#### 2.2.2. Current Candidates for Active Targeting

The unique features of cancer cells can be exploited in the development of targeted delivery system. For example, cancer cells often overexpress tumor antigens, carbohydrate-like structures, or growth factor receptors (e.g., epidermal growth factor receptor). Based on this concept, different types of ligands can be used as active targeting molecules such as antibody, polysaccharide, aptamer, peptide, transferrin, folate, and other small molecules [111]. Several examples of the targeting ligands bound to NPs and their corresponding targets are enlisted in Table 1. Choice of ligand depends on the cells to be targeted [112]. 

*Folic Acid*. Folic acid is a popular ligand that targets the folate receptors. Folate receptor is overexpressed in solid tumors such as breast, ovarian, lung, uterine, head, and neck cancers while normal tissues lack such abundance of the receptors. This could be an advantage for target ligands to seek out tumor cells [113]. Folate vitamin is an oxidized form of folate coenzyme having the predominant physiological form as “folic acid.” Folic acid has 30 times higher affinity for folate receptor than other folate derivatives. Folate conjugated molecules enter the cell through receptor mediated endocytosis process for targeted intracellular delivery of therapeutic agents. Drug conjugates such as liposomes, solid lipid NPs, polymeric NPs, polymers, and micelles are tailored with folate to subside the RES [114]. An experimental study for retinoblastoma by Das and Sahoo showed that the IC_50_ value for folate decorated Curcumin-Nutlin-3a loaded PLGA NPs was 8.6 times lower than folate unconjugated NPs. This dual drug loading was found to reverse multidrug resistance by downregulating multidrug resistance 1 (MRP-1) and lung resistance related proteins (LRP) [115].

*Transferrin*. Transferrin is a 78 kDa monomeric glycoprotein with the capacity of iron transportation in the body. Overexpression of transferrin receptors on cancer cells makes them an attractive target for its suitable ligand to deliver anticancer drugs into target cells. In particular, the abundance of transferrin receptors in brain glioma resulted in the development of the idea to treat glioblastoma using relative ligand-based therapies. Although transferrin receptor is also expressed in normal cells, overexpression of transferrin occurs in malignant transformations. During this transformation for further proliferation, cancer cells require more iron to synthesize DNA which results in overexpression of transferrin receptors [116]. 

*Angiogenesis Inhibitors*. Vascular endothelial growth factor receptors (VEGFR), integrin, matrix metalloproteinase receptor, and vascular cell adhesion molecule 1 (VCAM-1) are the targets for impairment of angiogenesis in tumor tissue. Integrin receptor is a good target for cancer treatment. Integrin *α*_*v*_*β*_3_ expression is found on activated endothelial cells, melanoma, and glioblastoma. A number of peptides and peptidomimetics have shown encouraging results for binding to *α*_*v*_*β*_3_ integrins. Thus, targeting the integrin receptors will allow the NPs to be internalized through the receptor based uptake mechanism. Peptide sequences like arginylglycylaspartic acid (RGD) has a high affinity for integrin *α*_*v*_*β*_3_ receptors overexpressed on angiogenic vasculatures. NPs loaded angiogenesis inhibitors have the advantage of overcoming physiological barriers and thereby diffuse the NPs through tumor irrespective of the tumor size. Some specific tumors and tumor endothelial cell receptors recognize and bind through the specific sequence of the peptide. For example, RGD grafted PEG-PLGA NPs has been found to enhance the targeting index as well as antitumor efficacy* in vivo*. The binding occurs between the RGD on NP and integrin overexpressed on malignant tumor cells [106, 117].

*Other Peptides*. Peptides have gained special interest in targeted delivery due to their small size, low immunogenicity compared to large proteins, high stability, and ease of conjugation with therapeutic NPs [118]. For example, cyclic-LABL peptide with the terminal functional amino group could be covalently attached to carboxyl terminal of the surfactant pluronic F-127 on the Doxorubicin loaded PLGA NP surface [48]. Other examples are listed in Table 1.

*Carbohydrates*. Another group of receptors is the C-type lectin receptors (CLR) that can recognize sugar molecules. There are several exogenous and endogenous CLRs on antigen presenting cells (APC) such as dendritic cells and macrophages. Haddadi et al. showed that carbohydrate mannan conjugated PLGA NPs enhanced dendritic cell maturation and stimulatory capacity compared to the nonconjugated formulation. These mannan conjugated NPs are targeted to the mannose receptor on the APCs [119].

*Biotin*. In addition to the aforementioned receptors for targeting purposes, biotin receptors are also overexpressed in many types of cancer. Studies have shown that biotin-free NPs result in a low degree of tumor reduction as well as survival in mice when compared with biotin conjugated nanoformulations. This might justify the decoration of PLGA NPs with biotin molecules [120].

*Antibodies*. Antibodies are the most diverse and broadly used targeting ligands that offer the advantages of high degree of specificity for the target tissues. However, antibodies should be linked to the NPs properly as impotent binding to NPs without specificity may impair the activity of antibody [121]. HER2, transferrin, and prostate-specific antigen (PSA) receptors are the most common targets for an antibody. Many monoclonal antibodies (mAb) have already been approved by FDA for targeted cancer treatment. Antibody fragments are also used as because they possess longer circulation time, smaller size, and low immunogenicity as well as the ability to overcome the steric hindrance for binding compared to full antibody. For example, antibody fragments (Fab) of anti-GD2 antibodies are used to target disialoganglioside GD2 of neuroblastoma cells. Application of Fab fragments is associated with increased cytotoxicity and binding ability for the target cancer cells of metastatic tumors. Due to their disadvantages, researchers are encouraged to use other ligands and small molecules [122]. Linking the cysteine residue of the fragmented antibody (Fab) to PEG chain results in increased circulation time of the modified NPs [123]. Both Fab and single chain variable fragment (scFv) lack the fragment crystallizable (Fc) domain that is present in the whole antibody responsible for complement activation. The lack of the Fc domain, in turn, reduces the immunogenicity of antibody fragments [124].

*Polysaccharides*. Targets for polysaccharides are still under exploration and investigation. However, polysaccharides such as chitosan, gelatin, and dextran are extensively used as biodegradable material to be incorporated in NPs [125]. Among these polysaccharides, hyaluronic acid (HA) is the natural polysaccharide that binds with HA receptors of target tumors. HA is a hydrophilic molecule that forms the shell and can be conjugated with the hydrophobic PLGA core. Studies showed that Docetaxel loaded PLGA core having HA in the outer surface contributed to a promising active targeting. HA creates a dynamic cloud of hydrophilic chains on the NP surface that repels plasma proteins. These targeted NPs showed better toxicity against breast cancer cells when compared with the nontargeted formulations [126]. Polysaccharide modification of NPs can help to avoid body's recognition mechanism. They contain various functional groups that facilitate self-assembly as well as drug conjugation. Using polysaccharides as core components modifies the polarity of the NP as well as colloidal stability. The use of either natural polysaccharides or their synthesized forms is possible. Polysaccharides can alter the surface charge of NPs, prolong the drug contact time with epithelium, enhance the cellular uptake, and increase bioavailability [127]. Chitosan is another naturally available linear polysaccharide that can easily react with other chemical reagents through its repetitive amine groups [128]. Often dual release of hydrophobic and hydrophilic drug could be performed using dual drug-release platform composed of chitosan film loaded with PLGA NPs [129].

*Aptamers*. Aptamers are single-stranded oligonucleotides having several advantages such as high targeting capacity, low production cost, simple synthesis process, and easy storage. Pegaptanib is an FDA approved aptamer bound with VEGF that blocks the interaction with VEGF receptor [130]. PSMA has also become a good target antigen in targeted drug delivery strategies. PSMA is expressed on some normal tissues, salivary glands, small intestine, and proximal renal tubules and overexpressed in prostate cancer cells (100–1000 times higher compared to normal tissues). PSMA is not a secretory protein like PSA and prostatic acid phosphatase (PAP). Rather, it has its own internalization function that increases when attached to anti-PSMA antibodies. Ligands such as scFv, antibodies, and aptamers have been used to target PSMA. Other prostate cancer antigens such as prostate cancer stem cell (CSC), HER2, and Mucin-1 as well as various receptors are ideal targets for prostate cancer treatment as well, but fewer studies have been performed to develop such targeted delivery systems [131]. Studies showed that Docetaxel loaded PLGA-PEG coblock polymer with terminal amine successfully functionalized with RNA aptamer for PSMA targeting was found more cytotoxic compared to the control group (aptamer free NPs). The hydrophilic PEG presents the carboxylic groups on the NP surface and minimizes the nonspecific uptake by the phagocytic system. However in a study, the tumor reduction capacity was shown to be successful in five mice out of seven along with 100% survival rate whereas nontargeted groups showed full tumor reduction with only 57% survival rate [75].

## 3. Current Approaches for Targeting NPs

There are several widely adopted approaches through which NPs could be intricately modified. The two commonly accepted methods include physical adsorption of hydrophilic polymer/drug onto NPs surface and chemical conjugation of ligand/drug to NPs. However, covalent binding method has been found to rule over the adsorption method since* in vivo *opsonins may compete with the adsorbed molecules onto NP surface. Therefore, better approaches have to be explored to structurally modify the end chains of biodegradable polymer [132]. This can be achieved either by priming the NP surface being introduced with a suitable functional group after preparation or by using the premodified polymeric strands for targeted NP preparation [42]. It is well known that the conjugating drug to the carrier in a binary delivery system rarely achieves specific targeting. However, engineered ternary NP consisting of a biocompatible polymer, drug molecule, and a ligand is essential for efficient targeted drug delivery system [17]. Various methods of tailoring targeting agents to NPs surface will be discussed in the following section.

### 3.1. Covalent Methods

#### 3.1.1. Antibody-NP Covalent Conjugation

**Figure 2:**
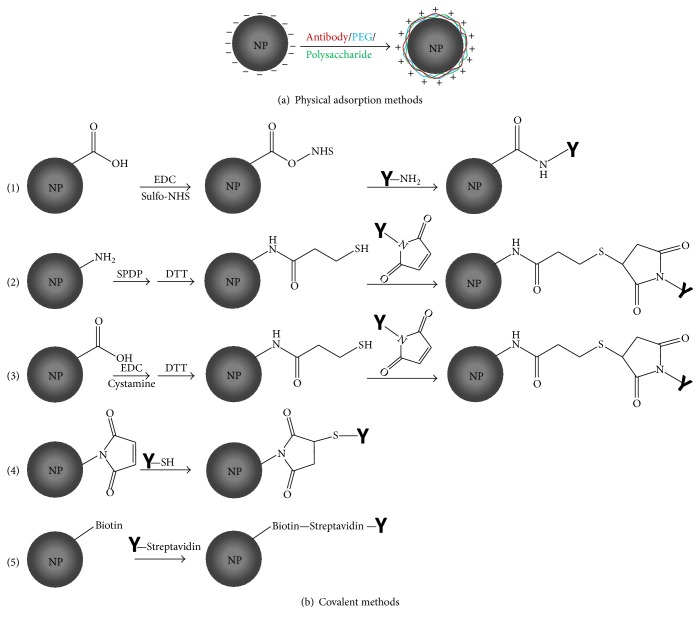
Schematic representation of ligand modified PLGA NPs by (a). Physical adsorption method and (b). Chemical conjugation methods, (1) carboxylic acid functionalized NPs form NHS-ester in presence of EDC and sulfo-NHS. NHS-ester reacts with primary amines to yield a stable amide bond, (2), (3), and (4). Thiol-maleimide reaction: activation of amine groups on NPs by SPDP and DTT followed by introduction of thiol groups that react with maleimide groups on the ligand, activation of carboxylic acid groups by EDC followed by introduction of thiol groups that react with maleimide groups on the ligand and maleimide coupling with activated thiol groups. (5) Noncovalent binding of biotin linked NP with ligand linked Streptavidin.

There are many covalent attachment methods being followed to chemically conjugate monoclonal or polyclonal antibodies with the prepared NPs using cross-linkers (Figure 2). In conjugation chemistry, utility of a spacer molecule is more than complementary for binding of mAb to the NP. Otherwise, the mAb could be self-polymerized and fail to recognize antibody binding sites on the NP [121]. Spacer group can be homobifunctional and heterobifunctional depending on the reactive groups at the end of the spacer arm. Homobifunctional cross-linkers are those having the same reactive chemical group at both ends, whereas these are different in heterobifunctional cross-linkers. The length of different spacer groups needs to be selected to avoid the occurrence of steric hindrance between drug and mAb [133]. Amino acids such as cysteine or cystamine having sulfhydryl group could be covalently attached to NPs resulting in the formation of thiolated NPs with free thiol groups accessible for antibodies or other drugs. 1-Ethyl-3-(3-dimethylaminopropyl) carbodiimide (EDC/EDAC), tris-carboxyethyl phosphine hydrochloride (TCEP), and dithio-DL-threitol (DTT) have also been used for the synthesis of thiolated NPs [134]. The following methods have been found more common for chemical conjugation utilizing various cross-linkers or spacer molecules.

*(i) Carbodiimide Chemistry for Conjugation*. Followed by the conjugation method based on carbodiimide chemistry, covalent amide bonds are theorized to form between the carboxylic group of NPs and amine group of the antibody or antibody fragment [135]. In brief, EDC reacts with the carboxylic acid groups of PLGA and activates their carbonyl moieties allowing them to be coupled with the functional amino group of antibody in the reaction mixture. NHS or its water-soluble analog (sulfo-NHS) is often included in EDC coupling protocols to improve the coupling efficiency [136]. This strategy was applied for targeted delivery of Docetaxel to cancer cell using an optimized formulation of anti-HER2 mAb decorated PLGA-PEG NPs where the free amine group of PEG was covalently attached to the carboxylic group of antibody [57]. Cirstoiu-Hapca et al. used a two-step carbodiimide reaction to thiolate the carboxylic groups on the surface of poly(DL-lactic acid) NPs. Then, the thiol groups on the Paclitaxel loaded NPs were linked to m-maleimidobenzoyl-N-hydroxy-sulfosuccinimide ester (sulfo-MBS) activated anti-HER2 mAb [137]. In another study, Pseudomonas exotoxin A loaded PLGA NPs were conjugated to fragmented SM5-1 mAb utilizing the carbodiimide chemistry. These NPs are injected into hepatocellular carcinoma SM5-1 binding protein overexpressing tumor xenograft mice model, resulting in tumor regression [138].

Carbodiimide conjugation is also used to obtain activated PLGA NPs preattached with biotin or PEG before treating with EDC/NHS. Cross-linker biotin binding proteins such as Avidin, Streptavidin, and NeutrAvidin can be attached to the biotinylated PLGA NP. In a comparative study with these three cross-linkers, it was found that NeutrAvidin showed higher specificity to the protein compared to Avidin and Streptavidin. Each cross-linker has four biotin binding sites to attach the biotinylated NPs that could lead to aggregation of NPs. Optimization of reagent's amount for biotinylation is the key step to overcome this aggregation issue [139]. Methods devoid of using EDC have also been included in this conjugation strategy due to similar reaction methodology. NHS activated palmitic acid (PA) reacts with positively charged Avidin. Various amount of PA-Avidin was added to PLGA NPs to quantify the surface density of Avidin on NPs. With increase in PA-Avidin amount, NP surface roughness was observed. This was attributed to the vesicle formation by PA that could spread onto the NP surface. The modified NPs showed less drastic morphological change in 21 days while plain NPs showed substantial change in morphology indicating the robustness of palmitoylated surface of NPs [140]. Increasing lipophilicity of the fatty acid linked to Avidin was found to increase the surface incorporation of Avidin to the NP. Maximum Avidin incorporation was found with Avidin-linoleic conjugate (approximately 100%). These NPs showed greater than 80% biotinylated horseradish peroxidase ligand binding to the NPs with the highest number (554 ± 72) of ligands per individual NP [141].

*(ii) Maleimide Chemistry for Conjugation*. The maleimide group reacts with sulfhydryl groups resulting in more selective and precise cross-linking. Combining sulfhydryl reactive groups with amine reactive groups through a maleimide heterobifunctional cross-linker provides better flexibility in antibody-NP conjugation [142]. Thiolated NPs were prepared via carbodiimide chemistry where cystamine could bind covalently with carboxylic group activated NPs. Sulfo-MBS was then used as a bifunctional cross-linker to bind the thiolated NPs and NeutrAvidin, which resulted in structurally modified NPs. The binding of model protein NeutrAvidin to the modified NP surface was higher than that of plain NP. NeutrAvidin-biotin binding ability was confirmed and proved no loss in protein activity during the attachment processes to the NPs [143]. In another study, thiol groups could be covalently introduced on PLA in a comparable way [144]. The NPs were formed via salting-out method containing about 25,000 thiol groups per NP. Covalent attachment of anti-HER2 and anti-CD20 antibody to the NPs through sulfo-MBS was performed and their targeting efficacy against target cancer cell lines was investigated. Optimization of the antibody type and spatial configuration rather than increasing its quantity on NP surface was found essential for internalization in human ovarian carcinoma cell (SKOV-3). Anti-HER2 modified NPs showed specific binding and internalization, whereas anti-CD20 modified NPs remained on the cell surface despite the higher number of anti-CD20 molecules bound to the NP surface [144]. While handling with thiol groups containing amine, it is difficult to introduce sufficient number of thiols since thiol groups are rapidly deteriorated in the solution. Modifications can be executed by introducing thiolating reagents such as N-succinimidyl-3-(2-pyridyldithio)-propionate (SPDP), N-succinimidyl S-acetylthioacetate (SATA), and a reducing agent such as DTT. This is an alternative way to introduce thiol groups on NPs followed by reaction with maleimide containing targeting agents [145].

*(iii) Click Chemistry for Conjugation*. Click chemistry is designated to run in mildly reactive conditions of aqueous solutions with 100% coupling efficiency. This coupling process offers some advantages over other bioconjugation strategies as it does not produce any undesirable side products such as dicyclohexylurea formed in carbodiimide reaction. The most common click reaction occurs between copper catalyzed 1,3 dipolar cycloaddition of azides and terminal alkynes known as CuAAC (copper catalyzed azide-alkyne cycloaddition). The 1,3-triazoles formed in this reaction are biocompatible entities approved by FDA to be used in drug formulation. Herein, the discrete PEG or propargyl-dPEG-NHS can be used as a spacer to react with an amine group of antibody. This azido spacer can be prepared in the presence of activating reagents such as EDC and sulfo-NHS at pH 6 [146]. Then the antibody linked azide group can react with terminal alkyne group containing fluorescent labelled PLGA-polymer blend. In recent studies, copper-free azide-alkyne cycloaddition is often used as it can reduce cytotoxicity* in vivo* [147].

*(iv) Conjugation Using Only Spacer*. In addition to the above conjugation methods, NPs can also be activated using a spacer molecule without any chemical modification which has been a new concept into practice for targeted delivery system. Therefore, manipulating NP preparation technique, the formation of unwanted intermediates during the procedure as formed in carbodiimide-amine and maleimide-thiol reactions can be avoided. Recently, Thamake et al. reported that noncovalent insertion of homobifunctional spacer Bis-sulfosuccinimidyl suberate (BS3) to PLGA NPs during the NP preparation can efficiently facilitate the covalent amide bond between the antibody and NP surface. In this one-step simple covalent attachment process, surface functionalization of NPs was performed by the formation of an amide linkage between the carboxylic group of BS3 and the amine group of the ligand upon hydrolysis of the terminal sulfo-NHS groups. The covalent bond between the antibody and BS3 was found with enhanced NP uptake by the cells [148].

#### 3.1.2. Polysaccharide-NP Covalent Conjugation

Chitosan could also form covalent bond between its amine group and carboxylic group of PLGA. The carboxylic moiety of PLGA NPs could be activated by EDC which reacts with amine group of chitosan. These NPs could achieve relatively constant release of the encapsulated anticancer agent mitoxantrone [92]. In another study, immobilization of thiol groups on the chitosan-NP surface was performed by covalent attachment of 2-iminothiolane to the free amino groups of chitosan. Covalent coupling of chitosan with 2-iminothiolane synthesized thiolated chitosan-4-thiobuthyimidine was observed with enhanced mucoadhesive properties. The resulting chitosan-modified NPs showed prolonged residence time on the small intestinal mucosa compared to chitosan-free PLGA NPs [91]. Similarly, carbodiimide activated carboxylic group of PLGA could be covalently attached to amino group of trimethylchitosan to form an amide bond. This trimethylated chitosan inclusion was found to form hydrophilic layer on NPs that triggers adsorptive-mediated transcytosis across the blood brain barrier (BBB) [90].

#### 3.1.3. PEG-NP Covalent Conjugation

Covalent attachment of PEG to the functional groups of preformed NP surface is also possible which can minimize the displacement of adsorbed copolymers by blood opsonins. PEG derivatives can form covalent bond with thiols, glycosylated proteins, and amino acids allowing PEG to act as the spacer between the NP and the modifying ligand [28].

### 3.2. Physical Adsorption Methods

In addition to covalent attachment methods, antibodies could also be attached to the NP surface following simple adsorption techniques, for instance, the development of cationic SMFv-polylys coated Paclitaxel loaded PLGA NPs. In this study, cationic polypeptide polylysine (polylys) was fused with SM5-1 scFv (SMFv) which was derived from SM5-1 mAb. The resulting positively charged fusion protein was tied with negatively charged Paclitaxel loaded PLGA NPs employing electrostatic interaction. The idea was to utilize the basic physicochemical properties of PLGA being negatively charged in neutral buffer where isoelectric point and electrostatic force strongly favor the adsorption of positively charged proteins on PLGA NP surface [59]. Following similar adsorption technique, transferrin and bovine serum albumin (BSA) were physically tagged with PLGA NPs where the protein ratio at 1/1 (w/w) was mixed with NPs (1 mg/ml concentration) with moderate shaking for 3 hours at room temperature [42]. However, the surface charge of PLGA NPs could be manipulated using different types of surfactants during NP preparation. Cetyltrimethylammonium bromide (CTAB) is an example of cationic surfactants which can be used in NP preparation to make positively charged NPs suitable for adsorbing plasmid DNA [149]. In another study to target human invasive ductal breast carcinoma, targeting ligands were physically linked to the NPs utilizing hydrophobic interaction between hydrophobic PLGA and hydrophobic part of the antibody molecule. In this study, slightly acidic phosphate buffered saline (PBS) of pH 5 was replaced with neutral buffer to facilitate the hydrophobic interaction [121]. Surface modification of hydrophobic NPs with hydrophilic PEG can also be performed by physical adsorption process [28]. Polysaccharides can form either monolayer or multiple layers around the NP core. A monolayer of cationic chitosan can be adsorbed onto PLGA chains of the NPs by electrostatic force [150]. Physical adsorption of more than one surface layer to PLGA NPs could be employed to target tumor cells efficiently. PLGA core could be physically coated with pluronic alone or a conjugate between heparin or chitosan to pluronic via urethane bond between the amine group of heparin or chitosan and hydroxyl group of pluronic [81].

## 4. Factors Controlling the Biological Response of Targeted NPs: Size, Surface Charge, and Storage Stability

Some physiological parameters including renal and hepatic filtration determine the biodistribution and therapeutic efficacy of NPs depending on their particle size and surface compositions. However, a very few experiments have been investigated with only focus on size and surface charge of targeted PEG/PLGA NPs. Among those, one study revealed that the particle size 100–200 nm demonstrated enhanced accumulation in tumors compared to the size of NPs < 100 and >200 nm. Moreover, 50% clearance was observed for NP's size over 400 nm in spleen [151]. In general, studies suggested that the polymeric NPs of 100 nm size range could be optimum for EPR effect, higher plasma concentration, and accumulation in tumor tissues [152]. It is also concurrently understood that the surface charge of NP plays a key role in the therapeutic activity and efficacy. For example, a highly negative and positive surface charge-based NPs show higher RES uptake and blood plasma protein aggregation. However, neutral to ±10 mV surface charge-based NPs exhibit an enhanced circulation [152–158]. Although targeting approach is an emerging aspect of NP-based therapy, it may change the required physicochemical properties of NPs. Some researchers have demonstrated that ligand-based targeted NPs resulted in limited tumor penetration, intracellular drug decomposition, and enhanced blood clearance due to changes in NP's physicochemical properties [67, 152, 159–161]. However, some of the researchers found promising results when the polymeric NPs were decorated with suitable ligands without changing their physicochemical properties.  Table 2 represents some recent studies for targeted PEG/PLGA NPs where the effects related to size and surface charge were comparatively discussed.

The results of these studies demonstrated the inevitability to control the size and surface charge of targeted NPs and could be a predictive guideline to formulate effective targeted therapeutic NPs before* in vivo* trials. However, a slight variation in size and surface charge of the NPs could make significant differences in both* in vitro* and* in vivo* studies.

The long-term storage of NPs is also an important parameter to scale up targeted nanoformulations. Irreversible aggregation may occur due to increased surface area of NPs when the formulations are stored for a longer duration. This can destabilize their physicochemical properties. The use of suitable cryoprotectant during lyophilization can be an approach to stabilize these PLGA NPs. Long-term storage of NPs with suitable cryoprotectant appeared to be stable without any polymer collapse or aggregation [162]. For example, Curcumin-loaded PLGA NPs were stable at room temperature and refrigerator even after 6 months of cryopreservation with 5% sucrose. It is anticipated that cryoprotectants provide a barrier on NP surface to prevent aggregation over the long-term storage [163]. It is important to have cryoprotected NPs as long-term storage leads to adsorption of moisture and reduction of *T*_*g*_ (glass transition temperature) below storage temperature, resulting in natural consequence of product collapse [164].

## 5. Biological Effects for Surface Functionalization of NPs

### 5.1. Surface Functionalization Using Antibodies

In order to target human invasive ductal breast carcinoma, antibody modified NPs following both covalent attachment and adsorption showed a comparative uptake in MCF-7 and MCF-10A neoT cells. These NPs were also found to be immune-specific since they were localized in antigen containing MCF-10A neoT cell lines rather than the antigen free CaCo-2 cells. Fluorescence microscopic study further confirmed that NPs with adsorbed mAbs showed higher binding to target cell than NPs with covalently linked mAbs. The hydrophilic surface of covalently attached NPs having defective antigen-binding domains could be responsible for hindering their uptake. In case of covalent binding, self-polymerization of the mAb could also take place and impede the binding ability of the mAb to its target leading to reduced biological activity [121]. In another study, anti-CD8 antibody was covalently attached to carboxylic ended PLGA through its amine group to prepare anti-CD8-PLGA NPs for targeting human CD8 antigen, a membrane protein which is an indicator of lymphoblastic leukemia cells. The internalization of anti-CD8-PLGA NPs in CD8 expressing cells occurred within one hour. But, the plain PLGA NP spreads randomly over the human embryonic kidney cell membrane surface for more than 24 hours [165].

When DTT is used for cleavage of disulfide bonds, its adsorption to the surface of NPs takes a longer period of time preventing the NP surface available for other conjugations. Besides, using TCEP shows the highest number of thiol groups to be introduced for covalent conjugation compared to DTT and EDAC [134]. Pseudomonas exotoxin A (PE) loaded PLGA NPs decorated with Fab fragments of a humanized SM5-1 mAb were more cytotoxic against hepatocellular carcinoma cell line than the exotoxin conjugated to the Fab fragment. Thus, they were more effective in inhibiting the tumor growth. PE-PLGA-SM5-1 NPs also showed lower immunogenicity than that of PE-SM5-1 conjugate. Additionally, the activity of the PE-SM5-1 conjugate was inhibited by anti-exotoxin neutralizing antibodies which resulted in low efficacy against the respective tumor cells. Therefore, the use of mutant immunotoxins could be replaced with exotoxin to be loaded into PLGA-SM5-1 NPs [138]. In addition, Kou et al. also prepared Paclitaxel loaded NPs and modified with SMFv linked to polylysine which showed specific binding affinity for SM5-1 binding protein expressed on the cancer cells followed by better cytotoxic effect than unmodified NPs. SMFv-polylysine acted as a ligand which retained the activity of parent antibody to recognize protein positive cells [59]. Interestingly, both SMFv-polylysine modified and unmodified NPs had similar cytotoxicity against cells having no SM5-1 binding protein expression. Moreover, biotinylated anti-mouse DEC-205 mAb attached to NP showed a proportional increase in both Interleukin-5 (IL-5) and IL-10 production with increasing antibody concentration to be used for conjugation [166]. Cytokine-based biotinylated antibody against CD4^+^ T lymphocytes linked to Avidin-Palmitate NPs promoted the potency of leukemia inhibitory factor cytokine 1000-fold over the soluble cytokine [167].

In another study, Chang et al. developed PLGA NPs decorated with BSA and transferrin following adsorption attachment procedure. Transferrin-PLGA NPs showed 20-fold higher endocytic competence for blood brain barrier than BSA-PLGA NPs. Filipin (caveolae-mediated endocytosis inhibitor) was used to show that transferrin-PLGA NPs were endocytosed via caveolae pathway. In contrast, uptake of BSA-PLGA NPs showed no reduction by filipin which was in support of its nonspecific endocytosis (adsorptive-mediated endocytosis) [42]. The PLGA polymers vary in their carboxylic acid group content. According to Scott et al., a higher density of carboxylic acid is associated with higher conjugation with antibody. Anti-siglec-7 polyclonal antibody was used to functionalize PLGA NPs of different contents of carboxylic acid group to target CD33 like siglec-7 receptor commonly expressed on most acute myeloid leukemia [168]. The density of polyclonal antibody on the NP surface was found variable with the amount of PLGA surface functional groups and their charge. Furthermore, the internalization of antibody was also influenced by the surface charge of the NPs. Fay et al. showed that the conjugation of AMG 655 death receptor 5 (DR5) specific antibodies (Conatumumab) to PLGA NPs was able to target DR5 receptors preferentially to induce subsequent apoptosis. This conjugation along with Camptothecin drug was able to show significant cytotoxic effect in colorectal cancer cells. Thus targeting the cancer cells along with chemotherapy was achieved [169].

### 5.2. Surface Functionalization Using Polysaccharide

A number of polysaccharide-decorated PLGA NPs have been summarized in Table 3. Particularly, it was found that covalently linked chitosan-PLGA NP showed comparatively higher drug release than PLGA NPs due to availability of microchannels on the surface. Mitoxantrone release was increased due to availability of hydroxy group on the surface that endowed more hydrophilic environment for drug release compared to pure PLGA NPs [92]. Yuan et al. prepared chitosan-PLGA NPs that could successfully entrap negatively charged siRNA within the chitosan core. Cationic chitosan interacted with anionic PLGA to form chitosan loaded PLGA NP. Due to high zeta potential the NPs were able to bind to more siRNA which resulted in enhanced endocytosis and silencing efficacy of green fluorescence protein into the cells [84]. Thiolation of chitosan-PLGA NPs was reported with excellent mucoadhesion property compared to the nonthiolated NPs. Moreover, the cationic chitosan-thiobutylamidine NPs showed strong mucoadhesion or successful thiolation with the anionic sialic acid and sulfonic acid substrate of mucus layer which was confirmed by an escalation of zeta potential from −22.91 mv to 24.75 mv. Therefore, thiolated chitosan could be a suitable approach when the mucoadhesion property is required for the formulations [91]. The mucoadhesive property was further investigated by Vllasaliu et al., where the model chitosan NPs could change the distribution of tight junction protein, zonula occludens-1 (ZO-1) in Calu-3 (human bronchial carcinoma) cells. However, they were not able to cross the tight junction part due to their large size (around 339 nm). Although the bronchial membrane permeability was not improved, NP containing drug could remain at the mucosa for a longer time [170]. This nanoparticulate system was persuasive for the possibility of controlled drug release and its prevention from enzymatic degradation.

For brain-targeted delivery, trimethylated chitosan covalently attached with drug coenzyme Q_10_ could be employed to enhance the uptake by cerebral endothelium. Trimethylated chitosan-covered particles could easily transport coenzyme Q_10_ through BBB which was not attainable with plain PLGA NPs [90]. In another study, hydrophobically modified dextran could be grafted to PLA and used to form NPs. Silylation of dextran chain was reported to decrease in chain polarity which could be restored by ring opening polymerization in order to impart amphiphilic properties of polymer [171]. When the PLA chain in the graft was short, the NP size became higher due to desorption and induced partial aggregation. In contrast, higher density NPs of smaller size were obtained when dextran was coated on PLA [172]. Additionally, NPs with lower size could be obtained when hydrophobically modified dextran with cholesterol was used. PLA-dextran-cholesterol NPs could adsorb fluorescein isothiocyanate (FITC) labelled BSA to their surface. The accumulation of these modified NPs in RES was more than PVA/PLA NPs having a hydrophilic layer that protected PVA stabilized PLA NPs from blood protein interaction [86]. To increase the NP accumulation in glioma cells, transferrin ligand was coupled on the PLA-dextran-cholesterol NPs where indomethacin was loaded as drug. The ligand-NP conjugate was treated with sodium tetraborate to prevent the leakage of indomethacin. FITC labelled NPs showed a comparative internalization into the glioma cancer cells within 4 hours [87]. Surface functionalization of modified PLA NPs was also performed with two bioactive ligands CD71 mAb and EGFR polyclonal antibody. Hydrophobically cholesterol modified dextran was obtained by esterification of dextran polyaldehyde and then attached with the antibodies at the functional aldehyde end groups. In brief, the amine group of antibody was linked with the aldehyde group of dextran and cholesterol facilitated the anchoring of the polysaccharide antibody conjugate to PLA NPs. The accumulation of NPs with one ligand in glioma cells was less compared to that of NPs with both ligands. The relative radioactivity in brain was found significant for two antibodies containing NPs which confirmed the overexpression of both antibody specific receptors on glioma cells [85]. Another experimental design was adopted to encapsulate water-soluble drug (vincristine sulfate) into PLGA NPs using anionic dextran sulfate sodium. Drug encapsulation efficiency was higher while using polysaccharide in the formulation which could be attributed to the generation of electrostatic interactions between polysaccharide and drug [89]. According to Vautheir et al., BSA adsorption in the diffuse shell of dextran could not be prevented due to ability of BSA to change its own conformation. However, adsorption of larger proteins such as fibrinogen could be controlled by the density of the dextran chains [173].

Chung et al. compared the adsorption of heparin/chitosan-pluronic (PEO-PPO-PEO) conjugate onto the PEG-PLGA core with the control standard PEG-PLGA-Pluronic NPs. They showed that chitosan-PLGA NP and heparin-PLGA NP exhibited more accumulation (2.4-fold) in tumor cells compared to plain PLGA NPs. But the limitation was determined by the comparative localization of these NPs in liver which justified the lack of tumor specificity of NPs [81]. This nonspecificity could be overcome by modifying NPs with ligands such as folate and aptamer [75, 174]. Other ligands such as folic acid and PEG-folic acid could be also covalently attached to act as coating for alginate-chitosan multilayer. The presence of alginate in outer layer enhanced the hydrophilicity that could resist BSA to interact with NPs. This indicated a lower susceptibility of the NPs to protein binding. But, the electrostatic interaction could interplay between chitosan and BSA when chitosan was present in the outer layer. However, 10–20% higher cell uptake was found when the chitosan-alginate NPs were coated with PEG-folic acid. Thus, functionalization with folate ligand as well as layer-by-layer employment of polysaccharide was found effective in targeting cancer [88].

### 5.3. Surface Functionalization Using PEG

In general, long-circulating NPs of size below 500 nm are able to accumulate in the tumor tissue and release the therapeutic agent through EPR effect [73]. Active targeting helps to select the target cancer cells rather than just accumulating passively at the cancer site. Use of small ligand-PEG conjugate may provide better biodistribution and enhanced recognition and effective internalization into the cancer cells [175]. Although the use of “stealth” approach of PEG is widespread, however, limitation lies in low drug loading efficiency if the drug is conjugated to the PEG chains [31]. Also, surface adsorbed PEG can desorb leaving holes in the surface where the circulating opsonins can bind. Additionally, it is sometimes difficult to ensure the covalent binding of PEG chains to the surface [26].  Table 4 enlists the representative examples of PLGA NP formulations decorated with PEG.

Besides, loading of PEG into PLGA NPs was found more effective than its surface adsorption to the particles in terms of bypassing the RES and increasing half-life during circulation. This could be attributed to the fact that loading of PEG resulted in more hydrophilic surface of NPs compared to the adsorbed PEG. PEG-loaded particles showed less liver accumulation (13.9%) whereas adsorbed PEG particles accumulation reached about 21.8% after 3 hours of injection [102]. In addition, molecular weight of PEG could be a crucial factor as higher molecular weight PEG was found to be significant in both formulations. Covalently grafted copolymeric PLA-PEG NPs were found to show less plasma protein adsorption on their hydrophilic surface compared to the PLA-PEG-PLA multiblock copolymeric NPs. This effect could be correlated to the different percentages of exposed PEG on the NPs surface. In an* in vitro* uptake study, the diblock copolymers resulted in lower internalization compared to the multiblock copolymer. This was explained by the relatively hydrophilic and less negative surface charge of the diblock polymeric NPs compared to the multiblock polymeric NPs [176]. There are several PEGs based polymeric NPs in clinical trials; for example, PEG-PLGA/PLA NP loaded with Docetaxel is in phase II for treatment of prostate cancer. Another phase III trial is on poly-amino acid-PEG-cisplatin derived micellar NP to treat lymphomas and solid tumors [177].

The mechanism of action of PEG correlates to the water molecules that form hydrogen bond with the ether oxygen of PEG which in turn repels protein interactions [175]. The flexibility of PEG chains creates a hydrated cloud that excludes interaction with opsonins and complement proteins. The distance between the PEG chain and NPs creates a conformation change that could act as a shield to repel any particles [178]. It was found that pretreatment of Avidin-coated PLGA NPs with biotin-PEG reduces nonspecific protein adsorption (<0.5 *μ*g/NP), whereas Avidin-coated NPs showed BSA adsorption of 1 *μ*g/NP. These PEG-modified NPs resulted in efficient release profiles of Doxorubicin with reduced cardiotoxicity. Taking serum creatine phosphokinase (CPK) as a measure of cardiotoxicity of Doxorubicin, it was found that the CPK was only 164 U/ml in PEGylated Doxorubicin loaded NPs compared to the free Doxorubicin (697 U/ml) treatment group [179].

The negative surface charge on NPs was found to activate the complement system proteins. It was reported that PEGylated PCL NPs recognition by MPS was assessed to be 45%, whereas non-PEGylated NPs showed about 90% MPS recognition just 5 minutes after injection [180]. NPs coated with PEG were also able to increase the bioavailability and pharmacokinetic profile of poorly water-soluble drug [181]. In another study, it was found that PEGylated NPs were able to accumulate in tumor graft within 7 hours; however PLGA NPs were not detectable in the respective tumor [182].

PEG present on NP's outmost layer can also be used to bind with ligands to target specific receptor which could be synergistic for NPs accumulation to the target site [183]. As a targeting ligand, folate is advantageous for its smaller size, simple conjugation chemistry, higher receptor affinity, lower immunogenicity, and limited expression in noncancerous tissues. PLGA-PEG-folate was found to have about 90% of entrapment efficiency of the anticancer drug (SN-38) while it was 77% for the non-PEGylated drug loaded NPs [184]. According to Farokhzad et al., nucleic acid ligand aptamer with amine (NH_2_) terminal could be covalently attached to PLA-PEG coblock having terminal carboxylic (COOH) group. It was reported that PEGylated-aptamer A10 NPs were competent to bind with the prostate cancer cell (77-fold higher than the control PEGylated NPs) [185]. Moreover, Docetaxel loaded PLGA-PEG-aptamer conjugate resulted in enhanced tumor size regression compared to empty NP, free Docetaxel, and Docetaxel loaded unmodified NPs in prostate cancer cell. A10 PSMA aptamer linked NPs were rapidly internalized in the tumor cell whereas lacking aptamer kept the particles onto the extracellular surrounding leading to rapid clearance [75]. Targeted delivery of cisplatin to prostate cancer cells was feasible via targeting PSMA using the aptamer attached to PLGA-PEG NPs.* In vitro* cytotoxicity assay showed that PSMA aptamer targeted cisplatin encapsulated NPs are 80 times more toxic than free cisplatin in the human prostate epithelial cells (LNCaP) [72]. When tested* in vivo*, this delivery system showed prolonged drug residence in blood with improved efficacy and tolerability over the free cisplatin [186]. In addition, biotinylated PEG-PLA block copolymers obtained via ring opening polymerization were successfully functionalized with transferrin that showed better internalization in C6 glioma cells compared to unmodified one. In a study, the intracellular uptake of transferrin modified NPs was 92.8% compared to about 68.3% uptake of plain PEG coated PLA NPs. The result indicated the advantage of ligand mediated active targeting over passive targeting [187]. Another study demonstrated that the hydrophilic nature of PEG was advantageous for binding of biotin which in turn resulted in significant binding of the fusion protein ligand linked to Streptavidin. This PLGA-PEG-ligand combination provided two advantages, one was the hindering of opsonins deposition and the other was the preferential uptake of ovalbumin loaded NPs by DEC-205 receptors [188].

## 6. Conclusion and Future Perspectives

Research on structure-activity relationship of NPs is an ongoing trend for developing structurally sound delivery systems. Newer and advanced technologies to characterize drug delivery systems are continuously inspiring researchers to drive for targeted delivery of drug in the body. Application of targeted NPs promises to lead the advances in new classes of therapeutics. A multifunctional delivery system with various therapeutic cargo could be effective against cancer [189]. It is important to investigate these types of formulations in clinical trials. However, the duration of clinical trials, the cost of treatment, and less number of patients are the limiting factors. It is encouraging that the preliminary data has potential, but more research is required to understand the basic principles that are involved with those specific targeting approaches, the disease conditions, disease stages, and the mechanism of action of the drugs. More detailed knowledge is needed to interpret the routes of drug delivery to assist them in achieving the therapeutic level of drug at the site of action. In future, strategies to treat resistant tumors, brain tumors, and metastasis, multipurpose delivery device could be utilized based on the results and targets mentioned in the review.

## Figures and Tables

**Table 1:** A brief overview and purposes of various actively targeted delivery systems using PLGA and PEG to their suitable targets.

Target cells/diseases/animal models	Targets	Targeted delivery system	Purpose of the study	Reference
*(I) Transferrin ligand*				

Leukemia (K562 cells)	Transferrin	Daunorubicin loaded PLGA-polylysine-PEG-transferrin	(i) To assess the antitumor efficacy of the delivery system with or without Daunorubicin/transferrin *in vitro* and *in vivo*	[41]

Brain capillary endothelial cells (BCEC) and astrocytes	Transferrin	PLGA NPs coated with bovine serum albumin/transferrin	(i) Evaluation of possible endocytosis mechanism for transferrin targeted brain drug delivery	[42]

Swiss albino mouse (female or male)	Transferrin	Lamotrigine loaded PLGA NPs	(i) Surface functionalization of NPs using transferrin and lactoferrin as ligand to deliver Lamotrigine to brain (ii) The purpose was to improve the biodistribution and pharmacokinetic profile of drug as well as reduced accumulation in nontargeted organs (kidney, lung, liver, spleen, heart) of the mouse	[43]

Pancreatic cancer cells	Transferrin	Bortezomib loaded PLGA NPs	(i) To study the targeting efficiency and capacity of transferrin targeted NPs	[44]

Breast cancer and glial cells	Transferrin	Curcumin/5-fluorouracil loaded magnetic PLGA NPs	(i) To identify the mechanism of cell death by the dual drug transferrin targeted NPs (ii) To identify the effect of magnetic hyperthermia to destroy cancer cells	[45]

Brain glioma cells	Transferrin	Paclitaxel and Doxorubicin loaded magnetic silica PLGA NPs	(i) To determine the transport efficiency through blood-brain barrier and target glioma cancer cells (ii) To investigate the therapeutic efficiency of targeted NPs in tumor bearing balb/c mice	[46]

*(II) Small peptides*				

*(1) Asparagine-glycine-arginine (NGR/RGD)*				

Human fibrosarcoma cell line (HT-1080) and human umbilical vein endothelial cells (HUVEC)	Aminopeptidase N (CD13)	Docetaxel loaded PLGA-PEG diblock copolymer NPs	(i) Exploration of the targeting potential of the drug loaded PEG-PLGA NPs *in vitro* cell lines (ii) Evaluation of hematologic toxicity, antitumor efficacy, nephrotoxicity and hepatotoxicity in balb/c mice	[47]

*(2) cLABL peptide against ICAM-1*				

A549 lung epithelial cells	Intercellular adhesion molecule-1 (ICAM-1)	Modified Pluronic® surfactant on PLGA NPs	(i) To find how NPs are targeted to lung epithelial cells via ICAM-1 to be internalized	[48]

*(3) Cyclo-arginine-glycine-aspartate (c-RGD) and combinations*				

Choroidal neovascularization (CNV) induced rat	Integrin/Transferrin	Antivascular endothelial growth factor intraceptor, Flt23K loaded PLGA-RGD/transferrin and/or PLGA-RGD-transferrin NPs	(i) To apply the targeted delivery of peptide modified PLGA NPs for the management of CNV, the cause of blindness due to macular degeneration	[49]

Human pancreatic cancer and human glioblastoma	Integrin *α*_*v*_*β*_3_	c-RGD-modified micelle-type PLGA-4-arm PEG	(i) To prove the use of multi-branched PLGA micelle as a diagnostic probe for pancreatic tumor detection	[50]

HUVEC cells	Integrin *α*_*v*_*β*_3_	Paclitaxel loaded PLGA-PEG NP or PEG-PCL (polycaprolactone) NP	(i) To investigate the effect of RGD peptide to target tumor endothelium and to see the antitumor efficacy of Paclitaxel	[51]

*(III) Folic acid and folic acid combinations with other ligands*				

Glioblastoma multiforme	Folate/lactoferritin	Folic acid or lactoferritin modified Etoposide encapsulated PLGA NPs	(i) Assessment of anti-tumor efficacy of Etoposide when encapsulated in the ligand-PLGA conjugate (ii) Identification of the expressions of folate and lactoferritin receptors in target cells	[52]

Human epidermal carcinoma cells	Folate	PLGA-folate and PLGA-RGD	(i) To confirm that surface modified NPs showed effective cellular uptake with no cytotoxicity	[53]

Human breast cancer cells (MCF-7)	Folate	Vincristine sulphate loaded PLGA-PEG-folate NPs or PLGA-PEG-cell penetrating peptide R7	(i) To investigate the cell uptake capacity of the ligand-drug-PLGA conjugate and ligand-PLGA conjugate (ii) To evaluate the tumor targeting and antitumor efficacy in *in vivo* model	[54]

Colorectal cancer	Folate	Folate modified Capecitabine loaded PLGA-PEG NPs and flate-PLGA-PEG NPs	(i) To prepare the two blends of NPs to evaluate their control release properties	[55]

Cervical tumor cells and human ovarian cancer cells	Folate	Folate modified Quercetin loaded PLGA-PEG NPs	(i) To test the cytotoxicity profile, targeting effect and cell uptake properties of the folate expressing cancer cells (ii) To demonstrate the active tumor targeting of folate bearing NPs	[56]

*(IV) Antibodies*				

SKBR-3 breast cancer cell	HER2	Anti-HER 2 trastuzumab antibody -modified Docetaxel-loaded PLGA	(i) To point out the feasibility of ligand conjugation strategy and demonstrate its efficiency in cell uptake and cytotoxicity	[57]

MCF-7 breast cancer cell	HER2	Anti-HER 2 trastuzumab antibody -modified human serum albumin NPs or gelatin NPs	(i) To observe the specific targeting of Herceptin conjugated NPs to Her2 overexpressed cells	[58]

Melanoma hepatocellular carcinoma and breast cancer cell	SM5-1 binding protein	Paclitaxel loaded PLGA linked with SM5-1 single chain antibody (scFv) derived from SM5-1 monoclonal antibody	(i) To develop the targeted NP system and examine their specific binding, cross-reactivity and internalization (ii) To evaluate the *in vitro* cytotoxicity effect of Paclitaxel loaded targeted NPs	[59]

Metastatic lesion of human prostatic adenocarcinoma	Androgen receptor (AR) and *β*-catenin	PSMA antibody conjugated PLGA-Curcumin NPs	(i) To generate an antibody conjugated targeted NP to target Ar/*β*-catenin in order to inhibit tumorigenesis	[60]

PSMA positive prostate cancer cell	PSMA positive cell surface	PLGA-PEG copolymer derived microbubble (MB) conjugated with urea based PSMA inhibitor molecular probe	(i) To establish the MB-molecular probe conjugate and confirm their selective binding to PSMA positive cells	[61]

*(V) Aptamer*				

PSMA on the surface of prostate cancer cell	PSMA	PLGA-PEG-Aptamer A10 triblock NPs	(i) To determine the optimum surface density of aptamer on the NP surface for maximum uptake by prostate cancer cell both *in vivo* and *in vitro*	[62]

Carbohydrate and polysaccharide				

Dendritic cell	Mannose receptor	Mannan-decorated PLGA NPs	(i) To incorporate mannose by covalent conjugation and adsorption method, compare the methods based on uptake of NPs by cells	[63]

Lung epithelium adenocarcinoma and human pulmonary microvascular endothelial cells	Hyaluronic acid (HA) receptor for HA NPs, for other systems it is not elucidated	Glycosaminoglycan such as heparin, HA, chondroitin sulfate and dermatan sulfate modified PLGA NPs	(i) Evaluation of the toxicity profile of the NP systems (ii) To validate the systems for lung cancer treatment	[64]

*(VI) Other ligands: nuclear localization signal (NLS) peptide*				

Breast cancer cell (MCF-7)	Cell nucleus	Doxorubicin loaded NLS-conjugated PLGA NPs	(i) To increase the concentration of Doxorubicin in cell nucleus via NLS targeted NPs (ii) To study the antiproliferative activity of targeted NPs and nontargeted NPs	[65]

**Table 2:** An overview of different targeting ligand decorated PLGA NPs with their preparation methods, ligands used, payloads, size, and zeta potential.

NPs type	NP preparation method	Targeting ligand	Loaded Materials	Stabilizer	Cell line/animal model	Average size (nm)	Zeta potential (mV)	Reference
PEG	Top-down strategic PRINT technique	Transferrin	—	—	HeLa, Ramos, H460, SK-OV-3, HepG2, LNCaP	(267 ± 49)–(292 ± 76)	(−35.6 ± 1.3)–(39.9 ± 1.7)	[66]

PEG-PCL	Dry-down method	Alexa Fluor 647 (AF647)	—	—	MDA-MB-468, MCF-7	(26.4 ± 0.7)–(60.9 ± 0.7)	(−5.1)–(−7.3)	[67]

PEG-PCL	Solvent evaporation	Angiopep-2	Paclitaxel	Sodium Cholate	U87 MG, BCECs	<100	(−3.08 ± 0.94)–(−3.28 ± 0.75)	[68]

PLGA	Nanoprecipitation	g7 Peptide	Loperamide, Rhodamine-123	Poloxamer 188	Tail vein in rats	140–180	−20	[69]

PLGA	Modified solvent extraction/evaporation	Trastuzumab	Paclitaxel	PVA	Caco-2, SK-BR-3	(293.8 ± 5.7)–(312.3 ± 8.2)	(−35.07 ± 1.68)–(−21.24 ± 2.11)	[70]

PLGA	Emulsion-solvent evaporation/extraction	Humanized anti-DC-SIGN (hD1)	FITC-TT peptide, DQ-BSA	PVA	Granulocytes, PBMCs	202 ± 4, 239 ± 14	(−28.6 ± 0.4), (−44.9 ± 1.8)	[71]

PLGA-PEG	Nanoprecipitation	A10 PSMA aptamer	Cisplatin	—	LNCaP, PC3	(131 ± 0.5)–(172 ± 3.4)	—	[72]

PLGA-PEG	Emulsion-solvent evaporation	Pep TGN	Coumarin-6	—	bEnd.3	(104.17 ± 3.45)–(121.46 ± 0.76)	(−24.43 ± 0.22)–(−18.25 ± 0.88)	[73]

PLGA-PEG	Solvent-diffusion	cRGD peptide	Doxorubicin	PEMA	MDA-MB-231, B16F10, MCF-7	(366.6 ± 3.1)–(423.0 ± 16.6)	(−18.9 ± 2.4)–(−51.7 ± 3.1)	[74]

PLGA-PEG	Nanoprecipitation	A10 2′-fluoropyrimidine RNA aptamers	Docetaxel	—	LNCaP	(153.3 ± 13.9)	−42 ± 1	[75]

PLGA–PEG	Emulsion-solvent diffusion	Folate binding protein	Docetaxel	PVA	SKOV3	(120 ± 5)–(216 ± 18)	(−6.27 ± 0.95)–(−12.2 ± 0.6)	[76]

PLGA-TPGS	Solvent extraction/evaporation	TPGS	Docetaxel	TPGS	Caco-2, MCF-7	(219.42 ± 5.24)–(253.51 ± 5.38)	(−21.87 ± 2.11)–(34.1 ± 4.28)	[77]

PLGA-TPGS	Solvent extraction/evaporation	Vitamin E TPGS-folate (TPGS-FOL)	Doxorubicin	—	MCF-7, C6 glioma	(324 ± 5)–(359 ± 10)	—	[78]

**Table 3:** Some examples of polysaccharide decorated PLGA NPs with their preparation methods, payloads, antibody conjugation methods, size, and zeta potential.

Shell and Core	Cargo/label	NP preparation method	Conjugation technique	Average size (nm)	Zeta potential (mV)	Reference
Chitosan/PEG functionalized PLGA core	Fluoresceinamine (FA)	O/W emulsification solvent evaporation	Covalent conjugation	329 (FA-chitosan-PLGA) 324 (FA-PEG-PLGA)	19.7 (FA-chitosan-PLGA) −38.1 (FA-PEG-PLGA)	[79]

Chitosan-PLGA engineered into mesenchymal stem cell	Paclitaxel, Curcumin	O/W emulsification solvent evaporation	Chemical conjugation	142	9.22	[80]

Heparin/chitosan-Pluronic on PEG-PLGA core	Tetramethyl rhodamine isocyanate	Nanoprecipitation/solvent diffusion	Chemical conjugation	144 (Hep-PLGA), 134 (Ch-PLGA)	−50 (Hep-PLGA), +38 (Ch-PLGA)	[81]

Chitosan coated on PLGA	siRNA	Double emulsification solvent evaporation	Surface adsorption	263.73	33.76	[82]

Lecithin/chitosan on PLGA core	Betamethasone-17-valerate (BSV)	Emulsion-diffusion evaporation	Ionic gelation	280.9 (BSV-chitosan-PLGA) 274 (BSV-chitosan-lecithin)	−5.62 (BSV-chitosan-PLGA) 40.8 (BSV-chitosan-lecithin)	[83]

6-Carboxyfluorescein-chitosan film on paclitaxel-PLGA core	siRNA	O/W emulsification solvent evaporation	Surface adsorption as film	204–543	+16.9–31.2	[84]

Dextran-cholesterol on PLA core	99mTc	Co-dialysis	Covalent attachment	119–201	—	[85]

Dextran-cholesterol on PLA core	Fluorescein isothiocyanate	O/W emulsification solvent evaporation	Covalent attachment	105	−11.9	[86]

Dextran-cholesterol-aldehyde on PLA core	Transferrin, fluorescein isothiocyanate	Co-dialysis method	Covalent attachment	100–150	−2.8	[87]

Alginate-chitosan on PLGA core	Folic acid or Folic acid-PEG	Emulsion solvent evaporation	Covalent attachment	—	—	[88]

Dextran-sulphate sodium and PLGA core	Vincristine sulphate	Nanoprecipitation	Self-assembly	128.3–133.0	(−10.6)–(−13.8)	[89]

Trimethylated chitosan- on PLGA core	Coenzyme Q_10_ and Coumarin-6	Nanoprecipitation	Covalent attachment	136.8–146.7	17.7–21	[90]

Chitosan-4-thiobutylamidine on PLGA core	Curcumin	Emulsification solvent evaporation	Covalent attachment	889.5	24.75	[91]

Chitosan on PLGA core	Mitoxantrone	Emulsion solvent evaporation	Adsorption and covalent attachment	291 (adsorption) 331 (covalent binding)	25.13 (adsorption), 23.01 (covalent binding)	[92]

**Table 4:** Some examples of PEG-modified PLGA NPs with preparation methods, targeting ligands, size, zeta potential, and applications.

Polymer	Targeting ligand	Conjugation method	Average size (nm)	Zeta potential (mV)	Applications	Reference
PEGylated PLGA	A10 aptamer	Covalent conjugation	188	N/A	Targeting human xenograft prostate cancer in mice	[93]

PLGA-PEG	—	Covalent conjugation	170	N/A	NPs encapsulating endostar slowed growth of tumor xenografts	[94]

PEGylated PLGA	cLABL	Covalent conjugation	244	−23.3	Targeting the vascular endothelium with upregulated ICAM-1	[95]

50/50 PLGA and Palmitate-Avidin	Biotinylated PEG, and horseradish peroxidase	Streptavidin-biotin noncovalent binding	170	−11.3	Diffusion of PEGylated particles cervical mucus was 3–10x higher than unmodified PLGA	[96]

PLGA-PEG	—	Covalent conjugation	148	1.84	Sustained release of 9-nitrocamptothecin	[97]

PLGA-PEG		Covalent conjugation	65–100	N/A	Sustained release of adriamycin	[98]

PLA-PEG		Covalent conjugation	952	Neutral	Reduced opsonization of NPs	[99]

PLGA and PEG-distearyl Phosphoethanolamine (PEGPE)		Coemulsification	20–40	−19.2	Higher Doxorubicin encapsulation efficiency, slower release rate, and rapid cellular uptake	[100]

PLGA-mPEG		Covalent conjugation	N/A	N/A	Reduction in protein adsorption on the surface films of PLGA-PEG (750 and 2000) compared to adsorption onto PLGA only	[101]

PLGA	PEG/poloxamer 407	Coincorporation or surface adsorption	189–225	(−16.1)–(−20.3)	Increased blood circulation half-life of NPs	[102]

PLGA-PEG di-block (15% PEG with 5 kDa)		Covalent conjugation	114	−2.8	Higher cellular uptake of formulations containing 15% of PEG compared to 5% and 10% PEG-PLGA formulations	[103]

## References

[1] Mousa S., Bharali D., Khalil M., Gurbuz M., Simone . (2009). Nanoparticles and cancer therapy: A concise review with emphasis on dendrimers. *International Journal of Nanomedicine*.

[2] Hochwald S. N. (2010). Molecular-targeted therapy for cancer and nanotechnology. *Cancer Nanotechnology*.

[3] Singh R., Lillard J. W. (2009). Nanoparticle-based targeted drug delivery. *Experimental and Molecular Pathology*.

[4] Cengelli F., Maysinger D., Tschudi-Monnet F. (2006). Interaction of functionalized superparamagnetic iron oxide nanoparticles with brain structures. *The Journal of Pharmacology and Experimental Therapeutics*.

[5] Ulbrich K., Holá K., Šubr V., Bakandritsos A., Tuček J., Zbořil R. (2016). Targeted drug delivery with polymers and magnetic nanoparticles: covalent and noncovalent approaches, release control, and clinical studies. *Chemical Reviews*.

[6] Mahapatro A., Singh D. K. (2011). Biodegradable nanoparticles are excellent vehicle for site directed in-vivo delivery of drugs and vaccines. *Journal of Nanobiotechnology*.

[7] Mundargi R. C., Babu V. R., Rangaswamy V., Patel P., Aminabhavi T. M. (2008). Nano/micro technologies for delivering macromolecular therapeutics using poly(d,l-lactide-co-glycolide) and its derivatives. *Journal of Controlled Release*.

[8] Grabnar P. A., Kristl J. (2011). The manufacturing techniques of drug-loaded polymeric nanoparticles from preformed polymers. *Journal of Microencapsulation*.

[9] Wang J. J., Zhao W. Z., Ren Z. X. (2011). Recent advances of chitosan nanoparticles as drug carriers. *International Journal of Nanomedicine*.

[10] Pejchal R., Doores K. J., Walker L. M. (2011). A potent and broad neutralizing antibody recognizes and penetrates the HIV glycan shield. *Science*.

[11] Nie S., Xing Y., Kim G. J., Simons J. W. (2007). Nanotechnology applications in cancer. *Annual Review of Biomedical Engineering*.

[12] Elsabahy M., Wooley K. L. (2012). Design of polymeric nanoparticles for biomedical delivery applications. *Chemical Society Reviews*.

[13] Mohamed F., van der Walle C. F. (2008). Engineering biodegradable polyester particles with specific drug targeting and drug release properties. *Journal of Pharmaceutical Sciences*.

[14] Danhier F., Feron O., Préat V. (2010). To exploit the tumor microenvironment: passive and active tumor targeting of nanocarriers for anti-cancer drug delivery. *Journal of Controlled Release*.

[15] Pelicano H., Martin D. S., Xu R.-H., Huang P. (2006). Glycolysis inhibition for anticancer treatment. *Oncogene*.

[16] Bazak R., Houri M., Achy S. E., Hussein W., Refaat T. (2014). Passive targeting of nanoparticles to cancer: A comprehensive review of the literature. *Molecular and Clinical Oncology*.

[17] Sinha R., Kim G. J., Nie S., Shin D. M. (2006). Nanotechnology in cancer therapeutics: bioconjugated nanoparticles for drug delivery. *Molecular Cancer Therapeutics*.

[18] Maeda H., Bharate G. Y., Daruwalla J. (2009). Polymeric drugs for efficient tumor-targeted drug delivery based on EPR-effect. *European Journal of Pharmaceutics and Biopharmaceutics*.

[19] Chipman S. D., Oldham F. B., Pezzoni G., Singer J. W. (2006). Biological and clinical characterization of paclitaxel poliglumex (PPX, CT-2103), a macromolecular polymer-drug conjugate. *International Journal of Nanomedicine*.

[20] Bae Y. H., Park K. (2011). Targeted drug delivery to tumors: myths, reality and possibility. *Journal of Controlled Release*.

[21] Bertrand N., Wu J., Xu X., Kamaly N., Farokhzad O. C. (2014). Cancer nanotechnology: the impact of passive and active targeting in the era of modern cancer biology. *Advanced Drug Delivery Reviews*.

[22] Siegler E. L., Kim Y. J., Wang P. (2016). Nanomedicine targeting the tumor microenvironment: Therapeutic strategies to inhibit angiogenesis, remodel matrix, and modulate immune responses. *Journal of Cellular Immunotherapy*.

[23] Vieira D. B., Gamarra L. F. (2016). Advances in the use of nanocarriers for cancer diagnosis and treatment. *Einstein (São Paulo)*.

[24] Jokerst J. V., Lobovkina T., Zare R. N., Gambhir S. S. (2011). Nanoparticle PEGylation for imaging and therapy. *Nanomedicine*.

[25] Moghimi S. M., Hunter A. C. (2000). Poloxamers and poloxamines in nanoparticle engineering and experimental medicine. *Trends in Biotechnology*.

[26] Owens D. E., Peppas N. A. (2006). Opsonization, biodistribution, and pharmacokinetics of polymeric nanoparticles. *International Journal of Pharmaceutics*.

[27] Roberts M., Bentley M., Harris J. (2002). Chemistry for peptide and protein PEGylation. *Advanced Drug Delivery Reviews*.

[28] Pasut G., Veronese F. M. (2012). State of the art in PEGylation: the great versatility achieved after forty years of research. *Journal of Controlled Release*.

[29] Gref R., Lück M., Quellec P. (2000). ‘Stealth’ corona-core nanoparticles surface modified by polyethylene glycol (PEG): Influences of the corona (PEG chain length and surface density) and of the core composition on phagocytic uptake and plasma protein adsorption. *Colloids and Surfaces B: Biointerfaces*.

[30] Siddiqui I. A., Adhami V. M., Ahmad N., Mukhtar H. (2010). Nanochemoprevention: Sustained release of bioactive food components for cancer prevention. *Nutrition and Cancer*.

[31] Alexis F., Pridgen E., Molnar L. K., Farokhzad O. C. (2008). Factors affecting the clearance and biodistribution of polymeric nanoparticles. *Molecular Pharmaceutics*.

[32] Song Q., Yao L., Huang M. (2012). Mechanisms of transcellular transport of wheat germ agglutinin-functionalized polymeric nanoparticles in Caco-2 cells. *Biomaterials*.

[33] Conde J., Dias J. T., Grazú V., Moros M., Baptista P. V., de la Fuente J. M. (2014). Revisiting 30 years of biofunctionalization and surface chemistry of inorganic nanoparticles for nanomedicine. *Frontiers in Chemistry*.

[34] Bazak R., Houri M., Achy S. E., Kamal S., Refaat T. (2014). Cancer active targeting by nanoparticles: a comprehensive review of literature. *Journal of Cancer Research and Clinical Oncology*.

[35] Shirshahi V., Shamsipour F., Zarnani A. H., Verdi J., Saber R. (2013). Active targeting of HER2-positive breast cancer cells by Herceptin-functionalized organically modified silica nanoparticles. *Cancer Nanotechnology*.

[36] Yu B., Tai H. C., Xue W., Lee L. J., Lee R. J. (2010). Receptor-targeted nanocarriers for therapeutic delivery to cancer. *Molecular Membrane Biology*.

[37] Karra N., Nassar T., Ripin A. N., Schwob O., Borlak J., Benita S. (2013). Antibody conjugated PLGA nanoparticles for targeted delivery of paclitaxel palmitate: Efficacy and biofate in a lung cancer mouse model. *Small*.

[38] An J., Teoh J. E., Suntornnond R., Chua C. K. (2015). Design and 3D printing of scaffolds and tissues. *Engineering Journal*.

[39] Brannon-Peppas L., Blanchette J. O. (2004). Nanoparticle and targeted systems for cancer therapy. *Advanced Drug Delivery Reviews*.

[40] Cho K., Wang X., Nie S., Chen Z., Shin D. M. (2008). Therapeutic nanoparticles for drug delivery in cancer. *Clinical Cancer Research*.

[41] Bao W., Liu R., Wang Y. (2015). PLGA-PLL-PEG-Tf-based targeted nanoparticles drug delivery system enhance antitumor efficacy via intrinsic apoptosis pathway. *International Journal of Nanomedicine*.

[42] Chang J., Jallouli Y., Kroubi M. (2009). Characterization of endocytosis of transferrin-coated PLGA nanoparticles by the blood-brain barrier. *International Journal of Pharmaceutics*.

[43] Lalani J., Patil S., Kolate A., Lalani R., Misra A. (2015). Protein-Functionalized PLGA Nanoparticles of Lamotrigine for Neuropathic Pain Management. *AAPS PharmSciTech*.

[44] Frasco M. F., Almeida G. M., Santos-Silva F., Do Carmo Pereira M., Coelho M. A. N. (2015). Transferrin surface-modified PLGA nanoparticles-mediated delivery of a proteasome inhibitor to human pancreatic cancer cells. *Journal of Biomedical Materials Research Part A*.

[45] Balasubramanian S., Girija A. R., Nagaoka Y. (2014). Curcumin and 5-fluorouracil-loaded, folate- and transferrin-decorated polymeric magnetic nanoformulation: a synergistic cancer therapeutic approach, accelerated by magnetic hyperthermia. *International Journal of Nanomedicine*.

[46] Cui Y., Xu Q., Chow P. K. H., Wang D., Wang C. H. (2013). Transferrin-conjugated magnetic silica PLGA nanoparticles loaded with doxorubicin and paclitaxel for brain glioma treatment. *Biomaterials*.

[47] Gupta M., Chashoo G., Sharma P. R. (2014). Dual targeted polymeric nanoparticles based on tumor endothelium and tumor cells for enhanced antitumor drug delivery. *Molecular Pharmaceutics*.

[48] Chittasupho C., Xie S.-X., Baoum A., Yakovleva T., Siahaan T. J., Berkland C. J. (2009). ICAM-1 targeting of doxorubicin-loaded PLGA nanoparticles to lung epithelial cells. *European Journal of Pharmaceutical Sciences*.

[49] Singh S. R., Grossniklaus H. E., Kang S. J., Edelhauser H. F., Ambati B. K., Kompella U. B. (2009). Intravenous transferrin, RGD peptide and dual-targeted nanoparticles enhance anti-VEGF intraceptor gene delivery to laser-induced CNV. *Gene Therapy*.

[50] Ding H., Yong K., Roy I. (2011). Bioconjugated PLGA-4-arm-PEG branched polymeric nanoparticles as novel tumor targeting carriers. *Nanotechnology*.

[51] Danhier F., Vroman B., Lecouturier N. (2009). Targeting of tumor endothelium by RGD-grafted PLGA-nanoparticles loaded with Paclitaxel. *Journal of Controlled Release*.

[52] Kuo Y.-C., Chen Y.-C. (2015). Targeting delivery of etoposide to inhibit the growth of human glioblastoma multiforme using lactoferrin- and folic acid-grafted poly(lactide-co-glycolide) nanoparticles. *International Journal of Pharmaceutics*.

[53] Park J., Brust T. F., Lee H. J., Lee S. C., Watts V. J., Yeo Y. (2014). Polydopamine-based simple and versatile surface modification of polymeric nano drug carriers. *ACS Nano*.

[54] Wang Y., Dou L., He H., Zhang Y., Shen Q. (2014). Multifunctional nanoparticles as nanocarrier for vincristine sulfate delivery to overcome tumor multidrug resistance. *Molecular Pharmaceutics*.

[55] Wei K., Peng X., Zou F. (2014). Folate-decorated PEG-PLGA nanoparticles with silica shells for capecitabine controlled and targeted delivery. *International Journal of Pharmaceutics*.

[56] El-Gogary R. I., Rubio N., Wang J. T.-W. (2014). Polyethylene glycol conjugated polymeric nanocapsules for targeted delivery of quercetin to folate-expressing cancer cells in vitro and in vivo. *ACS Nano*.

[57] Liu Y., Li K., Liu B., Feng S.-S. (2010). A strategy for precision engineering of nanoparticles of biodegradable copolymers for quantitative control of targeted drug delivery. *Biomaterials*.

[58] Wartlick H., Michaelis K., Balthasar S., Strebhardt K., Kreuter J., Langer K. (2004). Highly specific HER2-mediated cellular uptake of antibody-modified nanoparticles in tumour cells. *Journal of Drug Targeting*.

[59] Kou G., Gao J., Wang H. (2007). Preparation and Characterization of Paclitaxel-loaded PLGA Nanoparticles Coated with Cationic SM5-1 Single-chain Antibody. *BMB Reports*.

[60] Yallapu M. M., Khan S., Maher D. M. (2014). Anti-cancer activity of curcumin loaded nanoparticles in prostate cancer. *Biomaterials*.

[61] Sanna V., Pintus G., Bandiera P. (2011). Development of polymeric microbubbles targeted to prostate-specific membrane antigen as prototype of novel ultrasound contrast agents. *Molecular Pharmaceutics*.

[62] Gu F., Zhang L., Teply B. A. (2008). Precise engineering of targeted nanoparticles by using self-assembled biointegrated block copolymers. *Proceedings of the National Acadamy of Sciences of the United States of America*.

[63] Ghotbi Z., Haddadi A., Hamdy S., Hung R. W., Samuel J., Lavasanifar A. (2011). Active targeting of dendritic cells with mannan-decorated PLGA nanoparticles. *Journal of Drug Targeting*.

[64] Lamichhane S. P., Arya N., Ojha N., Kohler E., Prasad Shastri V. (2015). Glycosaminoglycan-Functionalized Poly-Lactide-Co-Glycolide nanoparticles: Synthesis, characterization, cytocompatibility, and cellular uptake. *International Journal of Nanomedicine*.

[65] Misra R., Sahoo S. K. (2010). Intracellular trafficking of nuclear localization signal conjugated nanoparticles for cancer therapy. *European Journal of Pharmaceutical Sciences*.

[66] Wang J., Tian S., Petros R. A., Napier M. E., DeSimone J. M. (2010). The Complex Role of Multivalency in Nanoparticles Targeting the Transferrin Receptor for Cancer Therapies. *Journal of the American Chemical Society*.

[67] Lee H., Fonge H., Hoang B., Reilly R. M., Allen C. (2010). The effects of particle size and molecular targeting on the intratumoral and subcellular distribution of polymeric nanoparticles. *Molecular Pharmaceutics*.

[68] Xin H., Jiang X., Gu J. (2011). Angiopep-conjugated poly(ethylene glycol)-co-poly(*ε*-caprolactone) nanoparticles as dual-targeting drug delivery system for brain glioma. *Biomaterials*.

[69] Tosi G., Fano R. A., Bondioli L. (2011). Investigation on mechanisms of glycopeptide nanoparticles for drug delivery across the blood-brain barrier. *Nanomedicine*.

[70] Sun B., Ranganathan B., Feng S.-S. (2008). Multifunctional poly(d,l-lactide-co-glycolide)/montmorillonite (PLGA/MMT) nanoparticles decorated by Trastuzumab for targeted chemotherapy of breast cancer. *Biomaterials*.

[71] Cruz L. J., Tacken P. J., Fokkink R. (2010). Targeted PLGA nano- but not microparticles specifically deliver antigen to human dendritic cells via DC-SIGN in vitro. *Journal of Controlled Release*.

[72] Dhar S., Gu F. X., Langer R., Farokhza O. C., Lippard S. J. (2008). Targeted delivery of cisplatin to prostate cancer cells by aptamer functionalized Pt(IV) prodrug-PLGA-PEG nanoparticles. *Proceedings of the National Acadamy of Sciences of the United States of America*.

[73] Torchilin V. (2011). Tumor delivery of macromolecular drugs based on the EPR effect. *Advanced Drug Delivery Reviews*.

[74] Wang Z., Chui W.-K., Ho P. C. (2009). Design of a multifunctional plga nanoparticulate drug delivery system: Evaluation of its physicochemical properties and anticancer activity to malignant cancer cells. *Pharmaceutical Research*.

[75] Farokhzad O. C., Cheng J., Teply B. A. (2006). Targeted nanoparticle-aptamer bioconjugates for cancer chemotherapy in vivo. *Proceedings of the National Acadamy of Sciences of the United States of America*.

[76] Esmaeili F., Ghahremani M. H., Ostad S. N. (2008). Folate-receptor-targeted delivery of docetaxel nanoparticles prepared by PLGA-PEG-folate conjugate. *Journal of Drug Targeting*.

[77] Chen H., Zheng Y., Tian G. (2011). Oral delivery of DMAB-modified docetaxel-loaded PLGA-TPGS nanoparticles for cancer chemotherapy. *Nanoscale Research Letters*.

[78] Zhang Z., Huey Lee S., Feng S.-S. (2007). Folate-decorated poly (lactide-co-glycolide)-vitamin E TPGS nanoparticles for targeted drug delivery. *Biomaterials*.

[79] Lautenschläger C., Schmidt C., Lehr C.-M., Fischer D., Stallmach A. (2013). PEG-functionalized microparticles selectively target inflamed mucosa in inflammatory bowel disease. *European Journal of Pharmaceutics and Biopharmaceutics*.

[80] Dai T., Yang E., Sun Y. (2013). Preparation and drug release mechanism of CTS-TAX-NP-MSCs drug delivery system. *International Journal of Pharmaceutics*.

[81] Chung Y.-I., Kim J. C., Kim Y. H. (2010). The effect of surface functionalization of PLGA nanoparticles by heparin- or chitosan-conjugated Pluronic on tumor targeting. *Journal of Controlled Release*.

[82] Jagani H. V., Josyula V. R., Palanimuthu V. R., Hariharapura R. C., Gang S. S. (2013). Improvement of therapeutic efficacy of PLGA nanoformulation of siRNA targeting anti-apoptotic Bcl-2 through chitosan coating. *European Journal of Pharmaceutical Sciences*.

[83] Özcan I., Azizoğlu E., Şenyiğit T., Özyazici M., Özer Ö. (2013). Comparison of PLGA and lecithin/chitosan nanoparticles for dermal targeting of betamethasone valerate. *Journal of Drug Targeting*.

[84] Yuan X., Shah B. A., Kotadia N. K., Li J., Gu H., Wu Z. (2010). The development and mechanism studies of cationic chitosan-modified biodegradable PLGA nanoparticles for efficient siRNA drug delivery. *Pharmaceutical Research*.

[85] Yuan X.-B., Kang C.-S., Zhao Y.-H. (2009). Surface multi-functionalization of poly(lactic acid) nanoparticles and C6 glioma cell trageting in vivo. *Chinese Journal of Polymer Science (English Edition)*.

[86] Ma W.-J., Yuan X.-B., Kang C.-S. (2008). Evaluation of blood circulation of polysaccharide surface-decorated PLA nanoparticles. *Carbohydrate Polymers*.

[87] Gu M.-Q., Yuan X.-B., Kang C.-S. (2007). Surface biofunctionalization of PLA nanoparticles through amphiphilic polysaccharide coating and ligand coupling: evaluation of biofunctionalization and drug releasing behavior. *Carbohydrate Polymers*.

[88] Zhou J., Romero G., Rojas E., Ma L., Moya S., Gao C. (2010). Layer by layer chitosan/alginate coatings on poly(lactide-co-glycolide) nanoparticles for antifouling protection and Folic acid binding to achieve selective cell targeting. *Journal of Colloid and Interface Science*.

[89] Ling G., Zhang P., Zhang W. (2010). Development of novel self-assembled DS-PLGA hybrid nanoparticles for improving oral bioavailability of vincristine sulfate by P-gp inhibition. *Journal of Controlled Release*.

[90] Wang Z. H., Wang Z. Y., Sun C. S., Wang C. Y., Jiang T. Y., Wang S. L. (2010). Trimethylated chitosan-conjugated PLGA nanoparticles for the delivery of drugs to the brain. *Biomaterials*.

[91] Grabovac V., Bernkop-Schnurch A. (2007). Development and in vitro evaluation of surface modified poly(lactide-co-glycolide) nanoparticles with chitosan-4-thiobutylamidine. *Drug Development and Industrial Pharmacy*.

[92] Chen H., Yang W., Chen H. (2009). Surface modification of Mitoxantrone-loaded PLGA nanospheres with chitosan. *Colloids and Surfaces B: Biointerfaces*.

[93] Cheng J., Teply B. A., Sherifi I. (2007). Formulation of functionalized PLGA-PEG nanoparticles for *in vivo* targeted drug delivery. *Biomaterials*.

[94] Hu S., Zhang Y. (2010). Endostar-loaded PEG-PLGA nanoparticles: in vitro and in vivo evaluation. *International Journal of Nanomedicine*.

[95] Zhang N., Chittasupho C., Duangrat C., Siahaan T. J., Berkland C. (2008). PLGA nanoparticle-peptide conjugate effectively targets intercellular cell-adhesion molecule-1. *Bioconjugate Chemistry*.

[96] Cu Y., Saltzman W. M. (2009). Controlled Surface Modification with Poly(ethylene)glycol Enhances Diffusion of PLGA Nanoparticles in Human Cervical Mucus. *Molecular Pharmaceutics*.

[97] Derakhshandeh K. (2010). Preparation and in vitro characterization of 9-nitrocamptothecin-loaded long circulating nanoparticles for delivery in cancer patients. *International Journal of Nanomedicine*.

[98] Davaran S., Rashidi M. R., Pourabbas B., Dadashzadeh M., Haghshenas N. M. (2006). Adriamycin release from poly(lactide-co-glycolide)-polyethylene glycol nanoparticles: Synthesis, and in vitro characterization. *International Journal of Nanomedicine*.

[99] Doiron A. L., Chu K., Ali A., Brannon-Peppas L. (2008). Preparation and initial characterization of biodegradable particles containing gadolinium-DTPA contrast agent for enhanced MRI. *Proceedings of the National Acadamy of Sciences of the United States of America*.

[100] Chu C.-H., Wang Y.-C., Huang H.-Y., Wu L.-C., Yang C.-S. (2011). Ultrafine PEG-coated poly(lactic-co-glycolic acid) nanoparticles formulated by hydrophobic surfactant-assisted one-pot synthesis for biomedical applications. *Nanotechnology*.

[101] Jeong J. H., Lim D. W., Han D. K., Park T. G. (2000). Synthesis, characterization and protein adsorption behaviors of PLGA/PEG di-block co-polymer blend films. *Colloids and Surfaces B: Biointerfaces*.

[102] Esmaeili F., Ghahremani M. H., Esmaeili B., Khoshayand M. R., Atyabi F., Dinarvand R. (2008). PLGA nanoparticles of different surface properties: preparation and evaluation of their body distribution. *International Journal of Pharmaceutics*.

[103] Pamujula S., Hazari S., Bolden G. (2012). Cellular delivery of PEGylated PLGA nanoparticles. *Journal of Pharmacy and Pharmacology*.

[104] Wang M., Thanou M. (2010). Targeting nanoparticles to cancer. *Pharmacological Research*.

[105] Arachchige M. C. M., Reshetnyak Y. K., Andreev O. A. (2015). Advanced targeted nanomedicine. *Journal of Biotechnology*.

[106] Jain A. K., Das M., Swarnakar N. K., Jain S. (2011). Engineered PLGA nanoparticles: an emerging delivery tool in cancer therapeutics. *Critical Reviews in Therapeutic Drug Carrier Systems*.

[107] Lammers T., Kiessling F., Ashford M., Hennink W., Crommelin D., Storm G. (2016). Cancer nanomedicine: is targeting our target?. *Nature Reviews Materials*.

[108] van der Meel R., Lammers T., Hennink W. E. (2017). Cancer nanomedicines: oversold or underappreciated?. *Expert Opinion on Drug Delivery*.

[109] Kumar Khanna V. (2012). Targeted Delivery of Nanomedicines. *ISRN Pharmacology*.

[110] Rajendran L., Knölker H.-J., Simons K. (2010). Subcellular targeting strategies for drug design and delivery. *Nature Reviews Drug Discovery*.

[111] Yang Z., Kang S.-G., Zhou R. (2014). Nanomedicine: De novo design of nanodrugs. *Nanoscale*.

[112] Muro S. (2012). Challenges in design and characterization of ligand-targeted drug delivery systems. *Journal of Controlled Release*.

[113] Narayanan S., Binulal N. S., Mony U., Manzoor K., Nair S., Menon D. (2010). Folate targeted polymeric 'green' nanotherapy for cancer. *Nanotechnology*.

[114] Zhao X., Li H., Lee R. J. (2008). Targeted drug delivery via folate receptors. *Expert Opinion on Drug Delivery*.

[115] Das M., Sahoo S. K. (2012). Folate decorated dual drug loaded nanoparticle: role of curcumin in enhancing therapeutic potential of nutlin-3a by reversing multidrug resistance. *PLoS ONE*.

[116] Tortorella S., Karagiannis T. C. (2014). Transferrin receptor-mediated endocytosis: A useful target for cancer therapy. *Journal of Membrane Biology*.

[117] Sultana S., Khan M. R., Kumar M., Kumar S., Ali M. (2013). Nanoparticles-mediated drug delivery approaches for cancer targeting: a review. *Journal of Drug Targeting*.

[118] Kamaly N., Xiao Z., Valencia P. M., Radovic-Moreno A. F., Farokhzad O. C. (2012). Targeted polymeric therapeutic nanoparticles: Design, development and clinical translation. *Chemical Society Reviews*.

[119] Haddadi A., Hamdy S., Ghotbi Z., Samuel J., Lavasanifar A. (2014). Immunoadjuvant activity of the nanoparticles' surface modified with mannan. *Nanotechnology*.

[120] Sah H., Thoma L. A., Desu H. R., Sah E., Wood G. C. (2013). Concepts and practices used to develop functional PLGA-based nanoparticulate systems. *International Journal of Nanomedicine*.

[121] Kocbek P., Obermajer N., Cegnar M., Kos J., Kristl J. (2007). Targeting cancer cells using PLGA nanoparticles surface modified with monoclonal antibody. *Journal of Controlled Release*.

[122] Torchilin V. P. (2010). Passive and active drug targeting: Drug delivery to tumors as an example. *Handbook of Experimental Pharmacology*.

[123] Chapman A. P. (2002). PEGylated antibodies and antibody fragments for improved therapy: A review. *Advanced Drug Delivery Reviews*.

[124] Allen T. M. (2002). Ligand-targeted therapeutics in anticancer therapy. *Nature Reviews Cancer*.

[125] Bolhassani A., Javanzad S., Saleh T., Hashemi M., Aghasadeghi M. R., Sadat S. M. (2014). Polymeric nanoparticles Potent vectors for vaccine delivery targeting cancer and infectious diseases. *Human Vaccines & Immunotherapeutics*.

[126] Huang J., Zhang H., Yu Y. (2014). Biodegradable self-assembled nanoparticles of poly(d,l-lactide-co-glycolide)/hyaluronic acid block copolymers for target delivery of docetaxel to breast cancer. *Biomaterials*.

[127] Myrick J. M., Vendra V. K., Krishnan S. (2014). Self-assembled polysaccharide nanostructures for controlled-release applications. *Nanotechnology Reviews*.

[128] Wang Y., Li P., Kong L. (2013). Chitosan-modified PLGA nanoparticles with versatile surface for improved drug delivery. *AAPS PharmSciTech*.

[129] Tada D. B., Singh S., Nagesha D. (2010). Chitosan film containing poly(D,L-Lactic-Co-Glycolic Acid) nanoparticles: A platform for localized dual-drug release. *Pharmaceutical Research*.

[130] Aslan B., Ozpolat B., Sood A. K., Lopez-Berestein G. (2013). Nanotechnology in cancer therapy. *Journal of Drug Targeting*.

[131] Barve A., Jin W., Cheng K. (2014). Prostate cancer relevant antigens and enzymes for targeted drug delivery. *Journal of Controlled Release*.

[132] Zhang L., Hou S., Mao S., Wei D., Song X., Lu Y. (2004). Uptake of folate-conjugated albumin nanoparticles to the SKOV3 cells. *International Journal of Pharmaceutics*.

[133] Lau A., Berube G., Ford C. H. (1995). Conjugation of doxorubicin to monoclonal anti-carcinoembryonic antigen antibody via novel thiol-directed cross-linking reagents. *Bioorganic & Medicinal Chemistry*.

[134] Nobs L., Buchegger F., Gurny R., Allémann E. (2003). Surface modification of poly(lactic acid) nanoparticles by covalent attachment of thiol groups by means of three methods. *International Journal of Pharmaceutics*.

[135] Byrne J. D., Betancourt T., Brannon-Peppas L. (2008). Active targeting schemes for nanoparticle systems in cancer therapeutics. *Advanced Drug Delivery Reviews*.

[136] Ikeda J., Sun Y.-L., An K.-N., Amadio P. C., Zhao C. (2011). Application of carbodiimide derivatized synovial fluid to enhance extrasynovial tendon gliding ability. *Journal of Hand Surgery*.

[137] Cirstoiu-Hapca A., Buchegger F., Bossy L., Kosinski M., Gurny R., Delie F. (2009). Nanomedicines for active targeting: Physico-chemical characterization of paclitaxel-loaded anti-HER2 immunonanoparticles and in vitro functional studies on target cells. *European Journal of Pharmaceutical Sciences*.

[138] Gao J., Kou G., Chen H. (2008). Treatment of hepatocellular carcinoma in mice with PE38KDEL type I mutant-loaded poly(lactic-co-glycolic acid) nanoparticles conjugated with humanized SM5-1 F(ab′) fragments. *Molecular Cancer Therapeutics*.

[139] Townsend S. A., Evrony G. D., Gu F. X., Schulz M. P., Brown R. H., Langer R. (2007). Tetanus toxin C fragment-conjugated nanoparticles for targeted drug delivery to neurons. *Biomaterials*.

[140] Fahmy T. M., Samstein R. M., Harness C. C., Saltzman W. M. (2005). Surface modification of biodegradable polyesters with fatty acid conjugates for improved drug targeting. *Biomaterials*.

[141] Park J., Mattessich T., Jay S. M., Agawu A., Saltzman W. M., Fahmy T. M. (2011). Enhancement of surface ligand display on PLGA nanoparticles with amphiphilic ligand conjugates. *Journal of Controlled Release*.

[142] Zhang F., Lees E., Amin F. (2011). Polymer-coated nanoparticles: A universal tool for biolabelling experiments. *Small*.

[143] Nobs L., Buchegger F., Gurny R., Allémann E. (2004). Poly(lactic acid) nanoparticles labeled with biologically active Neutravidin™ for active targeting. *European Journal of Pharmaceutics and Biopharmaceutics*.

[144] Cirstoiu-Hapca A., Bossy-Nobs L., Buchegger F., Gurny R., Delie F. (2007). Differential tumor cell targeting of anti-HER2 (Herceptin®) and anti-CD20 (Mabthera®) coupled nanoparticles. *International Journal of Pharmaceutics*.

[145] Shi M., Lu J., Shoichet M. S. (2009). Organic nanoscale drug carriers coupled with ligands for targeted drug delivery in cancer. *Journal of Materials Chemistry*.

[146] Li J., Ng C. K. (2012). *Methods for Nanoparticle Conjugation to Monoclonal Antibodies. Antibody-Mediated Drug Delivery Systems*.

[147] Wang C.-F., Mäkilä E. M., Kaasalainen M. H. (2014). Copper-free azide-alkyne cycloaddition of targeting peptides toporous silicon nanoparticles for intracellular drug uptake. *Biomaterials*.

[148] Thamake S. I., Raut S. L., Ranjan A. P., Gryczynski Z., Vishwanatha J. K. (2011). Surface functionalization of PLGA nanoparticles by non-covalent insertion of a homo-bifunctional spacer for active targeting in cancer therapy. *Nanotechnology*.

[149] Ataman-Önal Y., Munier S., Ganée A. (2006). Surfactant-free anionic PLA nanoparticles coated with HIV-1 p24 protein induced enhanced cellular and humoral immune responses in various animal models. *Journal of Controlled Release*.

[150] Guo C., Gemeinhart R. A. (2008). Understanding the adsorption mechanism of chitosan onto poly(lactide-co-glycolide) particles. *European Journal of Pharmaceutics and Biopharmaceutics*.

[151] Liu D., Mori A., Huang L. (1992). Role of liposome size and RES blockade in controlling biodistribution and tumor uptake of GM_1_-containing liposomes. *Biochimica et Biophysica Acta (BBA) - Biomembranes*.

[152] Li S.-D., Huang L. (2008). Pharmacokinetics and biodistribution of nanoparticles. *Molecular Pharmaceutics*.

[153] Xiao K., Li Y., Luo J. (2011). The effect of surface charge on in vivo biodistribution of PEG-oligocholic acid based micellar nanoparticles. *Biomaterials*.

[154] Semple S. C., Chonn A., Cullis P. R. (1998). Interactions of liposomes and lipid-based carrier systems with blood proteins: relation to clearance behaviour in vivo. *Advanced Drug Delivery Reviews*.

[155] Chonn A., Semple S. C., Cullis P. R. (1992). Association of blood proteins with large unilamellar liposomes in vivo: Relation to circulation lifetimes. *The Journal of Biological Chemistry*.

[156] Gessner A., Lieske A., Paulke B., Müller R. H. (2003). Functional groups on polystyrene model nanoparticles: Influence on protein adsorption. *Journal of Biomedical Materials Research Part A*.

[157] Levchenko T. S., Rammohan R., Lukyanov A. N., Whiteman K. R., Torchilin V. P. (2002). Liposome clearance in mice: the effect of a separate and combined presence of surface charge and polymer coating. *International Journal of Pharmaceutics*.

[158] Gessner A., Lieske A., Paulke B. R., Müller R. H. (2002). Influence of surface charge density on protein adsorption on polymeric nanoparticles: Analysis by two-dimensional electrophoresis. *European Journal of Pharmaceutics and Biopharmaceutics*.

[159] Andresen T. L., Jensen S. S., Jørgensen K. (2005). Advanced strategies in liposomal cancer therapy: Problems and prospects of active and tumor specific drug release. *Progress in Lipid Research*.

[160] Kirpotin D. B., Drummond D. C., Shao Y. (2006). Antibody targeting of long-circulating lipidic nanoparticles does not increase tumor localization but does increase internalization in animal models. *Cancer Research*.

[161] Chen W. C., Completo G. C., Sigal D. S., Crocker P. R., Saven A., Paulson J. C. (2010). In vivo targeting of B-cell lymphoma with glycan ligands of CD22. *Blood*.

[162] Ranjan A. P., Mukerjee A., Helson L., Vishwanatha J. K. (2012). Scale up, optimization and stability analysis of Curcumin C3 complex-loaded nanoparticles for cancer therapy. *Journal of Nanobiotechnology*.

[163] Grama C. N., Venkatpurwar V. P., Lamprou D. A., Ravi Kumar M. N. V. (2013). Towards scale-up and regulatory shelf-stability testing of curcumin encapsulated polyester nanoparticles. *Drug Delivery and Translational Research*.

[164] Fonte P., Soares S., Sousa F. (2014). Stability study perspective of the effect of freeze-drying using cryoprotectants on the structure of insulin loaded into PLGA nanoparticles. *Biomacromolecules*.

[165] Bicho A., Peça I. N., Roque A. C. A., Cardoso M. M. (2010). Anti-CD8 conjugated nanoparticles to target mammalian cells expressing CD8. *International Journal of Pharmaceutics*.

[166] Bandyopadhyay A., Fine R. L., Demento S., Bockenstedt L. K., Fahmy T. M. (2011). The impact of nanoparticle ligand density on dendritic-cell targeted vaccines. *Biomaterials*.

[167] Park J., Gao W., Whiston R., Strom T. B., Metcalfe S., Fahmy T. M. (2011). Modulation of CD4+ T Lymphocyte Lineage Outcomes with Targeted, Nanoparticle-Mediated Cytokine Delivery. *Molecular Pharmaceutics*.

[168] Scott C. J., Marouf W. M., Quinn D. J. (2008). Immunocolloidal targeting of the endocytotic siglec-7 receptor using peripheral attachment of siglec-7 antibodies to poly(lactide-co-glycolide) nanoparticles. *Pharmaceutical Research*.

[169] Fay F., McLaughlin K. M., Small D. M. (2011). Conatumumab (AMG 655) coated nanoparticles for targeted pro-apoptotic drug delivery. *Biomaterials*.

[170] Vllasaliu D., Exposito-Harris R., Heras A. (2010). Tight junction modulation by chitosan nanoparticles: Comparison with chitosan solution. *International Journal of Pharmaceutics*.

[171] Raynaud J., Choquenet B., Marie E. (2008). Emulsifying properties of biodegradable polylactide-grafted dextran copolymers. *Biomacromolecules*.

[172] Nouvel C., Raynaud J., Marie E., Dellacherie E., Six J.-L., Durand A. (2009). Biodegradable nanoparticles made from polylactide-grafted dextran copolymers. *Journal of Colloid and Interface Science*.

[173] Vauthier C., Persson B., Lindner P., Cabane B. (2011). Protein adsorption and complement activation for di-block copolymer nanoparticles. *Biomaterials*.

[174] Yoo H. S., Park T. G. (2004). Folate receptor targeted biodegradable polymeric doxorubicin micelles. *Journal of Controlled Release*.

[175] van Vlerken L. E., Vyas T. K., Amiji M. M. (2007). Poly(ethylene glycol)-modified nanocarriers for tumor-targeted and intracellular delivery. *Pharmaceutical Research*.

[176] Sant S., Poulin S., Hildgen P. (2008). Effect of polymer architecture on surface properties, plasma protein adsorption, and cellular interactions of pegylated nanoparticles. *Journal of Biomedical Materials Research Part A*.

[177] Anselmo A., Mitragotri S. (2016). Nanoparticles in the clinic. *Bioengineering & Translational Medicine*.

[178] Suk J. S., Xu Q., Kim N., Hanes J., Ensign L. M. (2016). PEGylation as a strategy for improving nanoparticle-based drug and gene delivery. *Advanced Drug Delivery Reviews*.

[179] Park J., Fong P. M., Lu J. (2009). PEGylated PLGA nanoparticles for the improved delivery of doxorubicin. *Nanomedicine: Nanotechnology, Biology and Medicine*.

[180] Shan X., Yuan Y., Liu C., Tao X., Sheng Y., Xu F. (2009). Influence of PEG chain on the complement activation suppression and longevity in vivo prolongation of the PCL biomedical nanoparticles. *Biomedical Microdevices*.

[181] Khalil N. M., do Nascimento T. C. F., Casa D. M. (2013). Pharmacokinetics of curcumin-loaded PLGA and PLGA-PEG blend nanoparticles after oral administration in rats. *Colloids and Surfaces B: Biointerfaces*.

[182] Diou O., Tsapis N., Giraudeau C. (2012). Long-circulating perfluorooctyl bromide nanocapsules for tumor imaging by 19FMRI. *Biomaterials*.

[183] Blanco E., Hsiao A., Mann A. P., Landry M. G., Meric-Bernstam F., Ferrari M. (2011). Nanomedicine in cancer therapy: Innovative trends and prospects. *Cancer Science*.

[184] Ebrahimnejad P., Dinarvand R., Jafari M. R., Tabasi S. A. S., Atyabi F. (2011). Characterization, blood profile and biodistribution properties of surface modified PLGA nanoparticles of SN-38. *International Journal of Pharmaceutics*.

[185] Farokhzad O. C., Jon S., Khademhosseini A., Tran T.-N. T., LaVan D. A., Langer R. (2004). Nanoparticle-aptamer bioconjugates: a new approach for targeting prostate cancer cells. *Cancer Research*.

[186] Dhar S., Kolishetti N., Lippard S. J., Farokhzad O. C. (2011). Targeted delivery of a cisplatin prodrug for safer and more effective prostate cancer therapy in vivo. *Proceedings of the National Acadamy of Sciences of the United States of America*.

[187] Ren W.-H., Chang J., Yan C.-H. (2010). Development of transferrin functionalized poly(ethylene glycol)/poly(lactic acid) amphiphilic block copolymeric micelles as a potential delivery system targeting brain glioma. *Journal of Materials Science: Materials in Medicine*.

[188] Raghuwanshi D., Mishra V., Suresh M. R., Kaur K. (2012). A simple approach for enhanced immune response using engineered dendritic cell targeted nanoparticles. *Vaccine*.

[189] Kang L., Gao Z., Huang W., Jin M., Wang Q. (2015). Nanocarrier-mediated co-delivery of chemotherapeutic drugs and gene agents for cancer treatment. *Acta Pharmaceutica Sinica B (APSB)*.

